# Stromal vascular fraction self-assembles vascularized osteogenic organoids with immunomodulatory functions

**DOI:** 10.1016/j.bioactmat.2025.10.030

**Published:** 2025-11-14

**Authors:** Jiazhou Wu, Ying He, Tao Qian, Zexian Liu, Yanbin Wu, Yazhou Li, Hongyu Jiang, Jianting Ye, Jia Lv, Biao Ma, Endong Luo, Jialiang You, Dingkai Wang, Yun Bai, Junming Zhang, Liang Zuo, Jiang Peng

**Affiliations:** a Institute of Orthopedics, The Fourth Medical Center of Chinese PLA General Hospital, Beijing Key Lab of Regenerative Medicine in Orthopedics, Key Laboratory of Musculoskeletal Trauma & War Injuries PLA, No. 51 Fucheng Road, Beijing, 100048, China; b Guizhou Medical University, Guizhou Province, 550004, China; c Graduate School of Chinese PLA General Hospital, No. 28 Fuxing Road, Beijing, 100853, China; d Department of Endocrinology, The First Affiliated Hospital of Jinzhou Medical University, Jinzhou, Liaoning, 121001, China

**Keywords:** Organoid, Bone tissue engineering, Stromal vascular fraction, Osteogenesis, Angiogenesis, Immunomodulation, 3D culture

## Abstract

Bone tissue engineering has enormous potential for treating complex orthopedic conditions such as Large Bone Defects (LBDs), delayed union or non-union fractures, and Osteonecrosis (ON). On the other hand, the adipose tissue-derived Stromal Vascular Fraction (SVF) comprises a heterogeneous population of cells, including Adipose-Derived Mesenchymal Stem Cells (ADSCs), Endothelial Progenitor Cells (EPCs), pericytes, and Hematopoietic Stem Cells (HSCs). Owing to its ease of acquisition, abundance, and the intrinsic involvement of its cellular components in bone repair, the SVF is presently considered a viable source for constructing osteogenic organoids. Herein, we leveraged the self-assembly capacity of SVF cells to construct spatially complex organoids with osteogenic, angiogenic, and immunomodulatory properties that mimic native tissues. Compared to spheroids from ADSCs alone, SVF cells exhibited natural tissue-like spatial organization, as well as superior osteogenic, angiogenic, and immunomodulatory potential, after two weeks of 3D spheroid culture. Furthermore, SVF-derived organoids showed distinct spatial architectures and functional profiles under both the growth and osteogenic induction conditions. Proteomic analysis and cytokine array profiling further revealed that SVF organoids were functionally enriched in the cytokine signaling and inflammatory/immune regulation pathways, with greater Extracellular Matrix (ECM) production observed under growth conditions compared to osteogenic induction. Moreover, *in vivo* experiments revealed that SVF organoids significantly accelerated fracture healing in nude mice. These findings present a simple and effective strategy for constructing multifunctional SVF-derived organoids, offering not only an innovative approach for bone tissue engineering but also a potential therapeutic avenue for clinical applications.

## Highlights

• Stromal vascular fraction (SVF) cells self-assemble into vascularized organoids with osteogenic and immune properties.
• SVF-derived organoids exhibit superior angiogenesis, osteogenesis, and cytokine signaling compared to ADSC spheroids.
• SVF organoids form physiologically relevant spatial architectures mimicking native bone tissue microenvironments.
• Without prior osteogenic induction, SVF organoids significantly accelerate fracture healing *in vivo*.
• This work provides a simple, scalable, and clinically translatable strategy for bone tissue engineering.

## Graphical abstract

**Figure:**
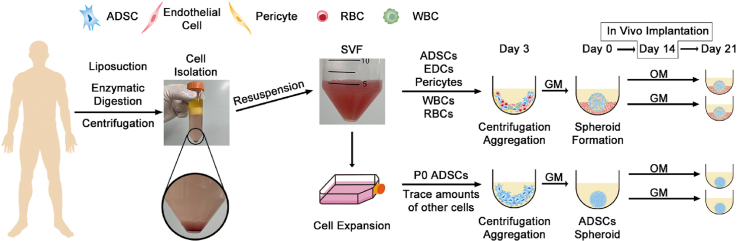

## 1 Introduction

Bone, a dynamic connective tissue that forms the skeletal framework, is essential for various physiological functions, including locomotion, hematopoiesis, calcium and phosphate storage, structural support, protection, and metabolic regulation [1]. Despite bone being one of the few organs in the human body with a robust regenerative capacity, severe conditions such as Large Bone Defects (LBDs), delayed union or nonunion fractures, and Osteonecrosis (ON)—often attributed to extensive tissue loss, chronic inflammatory stimulation, or imbalances in the local metabolic microenvironment—could still affect it, significantly impairing patients’ Quality of Life and Overall Survival (OS) [1,2]. Furthermore, although bone grafting is presently the first-line intervention for the aforementioned conditions, it has been reported to have several inherent limitations. Specifically, Allogeneic Bone Grafts (ABGs) often face challenges such as immune rejection and donor-recipient mismatch [3], while donor site availability, considerable surgical trauma, and prolonged recovery times could limit Autologous Bone Grafts (AutoBGs) [4]. In this regard, it is noteworthy that bone tissue engineering has recently emerged as a promising alternative, with significant therapeutic potential. It leverages 3-Dimensional (3D) cell culture systems, which more accurately mimic the native cellular microenvironment than traditional 2D cultures. These systems could promote cell-cell and cell-Extracellular Matrix (ECM) interactions, allowing the cells to organize into structured spatial arrangements within the 3D space. Besides preserving cellular morphology and functionality better, this phenomenon could provide a microenvironment conducive to the induction of specific biological functions, ultimately leading to organoid formation [[5], [6], [7]]. Organoids are 3-D structures generated *in vitro* from Pluripotent Stem Cells (PSCs) and Adult Stem Cells (ASCs), among other tissue-specific supporting cells. They often feature multiple organ-specific cell types, acquisition of certain physiological functions similar to the target organ, and native tissue-like spatial organization [8]. Besides their usefulness as platforms for drug screening and disease modeling, organoids have also been employed pervasively in tissue engineering for transplantation, wherein they could promote damaged organ repair and regeneration [8,9]. Therefore, organoid technology-based bone tissue engineering may represent a promising alternative to traditional bone grafting, providing a novel and effective therapeutic strategy for complex bone diseases.

Numerous studies have recently examined the construction of bone tissue engineering systems through the combination of Osteoprogenitor Cells (OPCs), osteoinductive biofactors, and biocompatible scaffold materials [[10], [11], [12]]. Mesenchymal Stem Cells (MSCs) have been widely employed as seed cells in bone tissue engineering, with Bone Marrow-derived MSCs (BMSCs) considered the prototypical MSCs for skeletal regeneration [13]. Despite the osteogenic potential of BMSCs in animal models of bone defects and some clinical trials, invasive harvesting procedures, associated trauma, and relatively low yield still limit their clinical applicability. Conversely, Adipose-Derived MSCs (ADSCs) are more readily accessible, abundant, and exhibit comparable multipotency, including the ability to differentiate into osteogenic, chondrogenic, and adipogenic lineages, making them a more viable alternative cell source for bone tissue engineering [[14], [15], [16]]. Owing to its high vascularization, which facilitates the delivery of oxygen, nutrients, and Growth Factors (GFs), native bone tissue is quite essential for bone development, regeneration, and remodeling [17]. According to research, avascular bone grafts integrate poorly with host tissues and lack adequate nutrient and oxygen supply, making them susceptible to necrosis [18]. In this regard, it is noteworthy that various strategies have been proposed to promote vascularization in osteogenic organoids. For instance, culture condition modulation could be leveraged to induce the simultaneous differentiation of MSCs into osteogenic and endothelial lineages. However, this approach requires precisely defined media compositions and complex culture protocols, and there are also challenges in maintaining robust osteogenic potential alongside a stable vascular network [19]. Additionally, Matrigel hydrogel and angiogenic GFs could be used to establish a vascular growth microenvironment. Nonetheless, due to Matrigel's derivation from mouse sarcoma cell lines and its ECM composition differing from the natural human cell-secreted ECM, there could be challenges in its clinical translation [20]. Furthermore, Human Umbilical Vein Endothelial Cells (HUVECs) could be co-cultured with MSCs to enhance bone regeneration and vascularization. Nevertheless, Endothelial Cells (ECs) and MSCs are often derived from different tissues and may vary among individuals, potentially limiting their use in autologous transplantation [21,22]. Based on these insights, developing better strategies would be imperative for enhanced vascularization in osteogenic organoids, significantly advancing the realms of bone tissue engineering and bone regeneration.

**Fig. 1:**
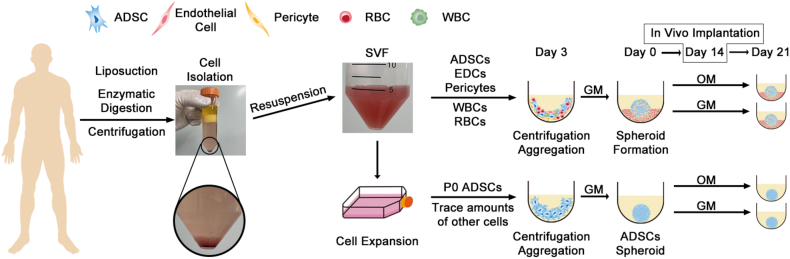
A schematic illustration of the experimental workflow. Abdominal adipose tissue was harvested from the same donor and processed to isolate the SVF. Meanwhile, primary ADSCs (P0 ADSCs) were obtained through an initial culture of SVF-derived cells. Both SVF and P0 ADSCs were seeded into ultra-low attachment 96-well U-bottom plates for spontaneous self-assembly into spheroids. After three days of spheroid formation, some spheroids were maintained in the Growth Medium (GM) without induction, while others were switched to the Osteogenic Medium (OM) for osteogenic induction. The spheroids were then cultured until day 21 post-formation. For *in vivo* transplantation in nude mice, spheroids were collected on day 14 after the initial formation.

The adipose tissue-derived Stromal Vascular Fraction (SVF), the initial extract from which ADSCs are obtained, comprises a heterogeneous population of cells, including ADSCs, Endothelial Progenitor Cells (EPCs), and pericytes, among other hematopoietic cells [23]. Compared to ADSCs, the SVF exhibited superior osteogenic and chondrogenic differentiation potential [24], as well as promising bone defect repair capabilities *in vivo* [25]. Additionally, several studies utilized endogenous ECs within the SVF to construct vascularized adipose organoids [7,26,27]. These findings collectively suggest that the SVF could be an ideal cell source for constructing vascularized osteogenic organoids. Moreover, the role of inflammation and Immune Microenvironment (IME) modulation in bone healing has recently gained increasing attention. During the early stages of fracture repair, M1 macrophages may secrete pro-inflammatory cytokines, initiating a cascade of responses, which ultimately remove necrotic tissues and promote fracture healing [28,29]. Additionally, M2 macrophages could secrete GFs in the mid-to-late stages of healing, promoting bone remodeling [28,30]. Moreover, since the SVF naturally contains various immune cells, including macrophages, it offers several physiological benefits that may enhance its applicability in bone tissue engineering [31]. In other words, SVF-based constructs may possess a more biomimetic and functional niche for osteogenic organoid development. Herein, we leveraged the self-assembly properties of SVF cells to construct organoids with a physiologically relevant spatial architecture, as well as osteogenic, angiogenic, and immunomodulatory properties. Primary ADSC spheroids derived from the same donor were used as controls, and the effects of osteogenic induction and non-induction culture conditions on organoid development were systematically compared (Fig. 1). We also assessed the differences in spatial organization and biological functionality *in vitro* between SVF- and ADSC-derived spheroids under both culture conditions. Furthermore, the therapeutic efficacy of the different spheroid types in promoting bone repair was evaluated using an *in vivo* femoral fracture model in nude mice. Overall, we found SVF to be an ideal cell source for constructing and maintaining osteogenic organoids, presenting a novel strategy and promising alternative for bone tissue engineering applications.

## 2 Materials and methods

### 2.1 Ethical approval

Adipose tissue samples were collected from 50 male patients [aged: 20–50 years; mean age: 34.96 years; Body Mass Index (BMI): 26.06 ± 3.84 kg/m^2^] who underwent liposuction at the Fourth Medical Center of the Chinese PLA General Hospital. All patients had no history of metabolic or endocrine disorders, alcohol abuse, smoking, or use of medications affecting glucose or lipid metabolism. Each sample was handled separately without pooling. The Ethics Committee of the Chinese PLA General Hospital approved the study protocol (Approval No. 2023KY088-KS001), and all participants provided informed consent.

### 2.2 Isolation of SVF and ADSCs, and spheroid culture

Adipose tissues were processed within 6 h of collection, with each sample divided into two portions: 5 mL for P0 ADSC culture and the remaining for SVF isolation. For SVF preparation, adipose tissue was digested with 0.1 mg/mL Liberase™ (Roche, Switzerland) in a 37 °C water bath for 45 min with intermittent shaking. The digested tissue was then centrifuged at 1500 rpm for 10 min, before filtering through a 70 μm cell strainer to obtain cell pellets. Subsequently, the cell pellets were resuspended in human MSC GM (MSC-T4, BASO, USA), counted, and seeded at a density of 3 × 10^5^ cells/well into ultra-low attachment 96-well U-bottom plates (Corning, USA). This seeding density was determined based on results from preliminary optimization experiments. Notably, spheroids were formed after 3 days of culture (designated as day 0). To establish the SVF GM and SVF OM groups, the spheroids were either maintained in GM (MSC-T4) or switched to OM (OriCell, China), respectively. For P0 ADSC preparation, a designated portion of adipose tissue was digested and centrifuged as outlined above. The cell pellets were then seeded into T75 culture flasks, where adherent cells were expanded under standard conditions. At 80–90 % confluence, the cells were detached using trypsin and seeded into 96-well U-bottom plates at a similar density (3 × 10^5^ cells/well). After 3 days (considered day 0) of spheroid formation, the spheroids were either cultured in GM or switched to OM, creating the ADSC GM and ADSC OM groups, respectively. Finally, the four groups (SVF GM, SVF OM, ADSC GM, and ADSC OM) were compared. Cells from the same donor were used in all comparisons between SVF and ADSC spheroids. Moreover, *in vitro* and *in vivo* experiments were repeated at least three times and at least four times, respectively.

### 2.3 Phenotypic characterization of SVF and P0 ADSCs

After preparing single-cell suspensions of SVF and P0 ADSCs (1 × 10^6^ cells/mL), 0.2 mL of each suspension was separately aliquoted into individual EP tubes. For comprehensive phenotypic analysis, a mixture of antibodies against CD31, CD34, CD45, CD73, CD90, and CD146 was added to Tube A, while Tubes B-G were stained with individual antibodies for compensation controls. Tube H served as the PBS blank control. Furthermore, each tube received 1 μL of viability dye and was incubated in the dark for 30 min. After washing with Phosphate Buffered Saline (PBS), the samples were analyzed using a flow cytometer (BD LSRFortessa Special Order Research Product, USA), and the collected data were processed using FlowJo software. All tests were repeated four times (n = 4/group).

For macrophage phenotyping within SVF, single-cell suspensions were prepared at a concentration of 6 × 10^6^ cells/mL. Then, 100 μL of the cell suspension was mixed with 100 μL of PBS and 1 μL of viability dye. This mixture was incubated with antibodies against CD45, CD11b, CD14, CD68, HLA-DR, CD86, and CD206 in the dark at room temperature for 30 min. After PBS washing, the samples were analyzed on the same flow cytometer, and data were processed with FlowJo software. Each group was analyzed in quadruplicate (n = 4/group).

### 2.4 Spheroid formation and morphological analysis

Spheroid formation from SVF and P0 ADSCs was monitored every 12 h using a stereomicroscope (Nikon DS-Ri2, Japan) until day 3, when it was considered complete (designated as day 0). Subsequently, the spheroids were divided into four groups: SVF GM, SVF OM, ADSC GM, and ADSC OM, with bright-field images captured on days 0, 7, 14, and 21 post-formation using an optical microscope (Nikon 2000, Japan). Finally, the spheroid area was measured using NIS-Elements software, with 30 spheroids analyzed per group at each time point.

### 2.5 Cell viability assays

Cell viability was assessed using the PrestoBlue reagent (Thermo Fisher Scientific, USA) on days −3, −2, and −1 before spheroid formation, and on days 0, 7, 14, and 21 post-formation. Briefly, PrestoBlue was added to the culture wells at a final concentration of 10 %, and incubated at 37 °C for 1 h. After collecting the supernatants, absorbance was measured at 570 nm and 600 nm, with the viability normalized to the 600 nm reference wavelength. Each group was tested three times at each time point.

### 2.6 Proliferation and apoptosis staining

On days 0, 7, 14, and 21 post-formation, spheroids were collected from each group and then fixed, dehydrated, embedded, and cryosectioned into 5 μm-thick slices. For Ki67 proliferation staining, the sections first underwent antigen retrieval and blocking with Bovine Serum Albumin (BSA), and then incubated with anti-Ki67 primary antibody (Abcam ab15580, USA; 1:200) at 37 °C for 2 h. After washing, Horseradish Peroxidase (HRP)-conjugated secondary antibodies were applied, followed by TSA fluorescent labeling and DAPI nuclear counterstaining. Subsequently, fluorescent images were captured using a fluorescence microscope, and the percentage of Ki67-positive cells was quantified using ImageJ software. For TUNEL apoptosis staining, the sections were first permeabilized using a permeabilization solution (Beyotime P0097, China) and then incubated with TUNEL reaction mixture (Beyotime C1090, China) at 37 °C in the dark for 1 h. Following that, DAPI was added for nuclear counterstaining. Finally, fluorescent images were acquired, and the proportion of TUNEL-positive cells was analyzed using ImageJ software. Each group was tested three times at each time point.

### 2.7 Hematoxylin and eosin (H&E) histological staining

On days 0, 7, 14, and 21 post-formation, spheroids from each group were collected and then fixed, dehydrated in sucrose, embedded in OCT compound, and cryosectioned. After removing excess OCT, the sections were stained using an H&E staining kit (Solarbio G1121, China), dehydrated, and mounted with neutral resin. Finally, images were captured using a panoramic confocal digital slide scanner (DynaMax Biotech, China). Each group was analyzed three times at each time point.

### 2.8 Cytoskeletal morphology analysis

On days 0, 7, 14, and 21 post-formation, samples were collected from each group and then fixed with 4 % Paraformaldehyde (PFA) for 1 h, dehydrated in sucrose, embedded in OCT, and cryosectioned into 5-μm slices. After antigen retrieval and blocking, the sections were incubated with an anti-α-Tubulin primary antibody (Abcam ab7291, 1:200 dilution) at 37 °C for 2 h. After washing, HRP-conjugated secondary antibodies were added before performing TSA fluorescent signal amplification and DAPI counterstaining. Finally, cytoskeletal structures within the spheroids were visualized using a fluorescence microscope. Each group was analyzed three times at each time point.

### 2.9 Transmission electron microscopy (TEM) for ultrastructure analysis

On day 14 post-formation, spheroids from each group were collected and fixed in 2.5 % Glutaraldehyde (GA) for 24 h. After washing with PBS, the samples were fixed with 1 % osmium tetroxide, dehydrated using a graded ethanol series, and infiltrated with a gradient of acetone and embedding resin. The samples were then polymerized at 70 °C. Ultrathin sections (70–90 nm) were then prepared and stained with uranyl acetate and lead citrate. Finally, ultrastructural features of the spheroid interior were observed using a transmission electron microscope.

### 2.10 Multiplex immunofluorescence (IF) Co-localization staining

At days 0, 7, 14, and 21 after spheroid formation, the samples were collected and cryosectioned. A multiplex IF staining kit (Boster PTSA-67, China) was then used for iterative multicolor labeling, involving antigen retrieval, BSA blocking, primary antibody incubation (1:200), HRP-conjugated secondary antibody incubation, TSA signal amplification, and repeated antigen retrieval for up to five sequential rounds using 520Plus, 570Plus, 620Plus, 690Plus, and 480Plus fluorophores. Subsequently, DAPI was added for nuclear staining. The primary antibodies included: CD44 (Abcam ab254530, USA), CD31 (Abcam ab9498, USA), PDGF-R (Abcam ab32570, USA), CD68 (Abcam ab955, USA), Vinculin (Abcam ab129002, USA), VEGF (Abcam ab52917, USA), RUNX2 (Abcam ab192256, USA), COL1 (Abcam ab138492, USA), and SOX9 (Abcam ab185966, USA). The slides were then mounted before capturing images using a whole-slide multispectral imaging system (Akoya, USA). Finally, marker expression ratios or mean fluorescence intensity were analyzed quantitatively using ImageJ software. Each group was examined three times at each time point.

### 2.11 Multilineage differentiation induction and histological staining

Osteogenic, chondrogenic, and adipogenic induction protocols were performed to evaluate the multilineage differentiation potential of SVF- and ADSC-derived spheroids. From day 3 post-formation, the spheroids were divided into induction groups and cultured in OM, Chondrogenic Medium (CM), or Adipogenic Medium (AM) (all from OriCell, China). Corresponding control groups were maintained in standard GM (MSC-T4, BASO, USA). All media were half-exchanged daily. On days 7, 14, and 21, spheroids were collected from each group for analysis. Before staining, all samples were fixed, dehydrated, embedded in OCT compound, and cryosectioned. Osteogenic differentiation was then assessed using Alkaline Phosphatase (ALP) staining (Beyotime C3206, China) and Alizarin Red S (ARS) staining. On the other hand, chondrogenic differentiation was evaluated using Alcian Blue Staining (ABS), while adipogenic differentiation was assessed using Oil Red O Staining (OROS). All experiments were repeated three times for each group at each time point.

### 2.12 Quantitative real-time polymerase chain reaction (qRT-PCR)

First, samples were collected from the SVF-GM, SVF-OM, ADSC-GM, and ADSC-OM groups on day 14 after spheroid formation. Subsequently, Red Blood Cells (RBCs) in the SVF groups were lysed using RBC lysis buffer (Solarbio R1010, China). Total RNA was then extracted using the FastPure Cell/Tissue Total RNA Isolation Kit V2 (Vazyme, China), and subsequently reverse-transcribed using the HiScript III All-in-one RT SuperMix Perfect for qPCR kit (Vazyme, China). Following that, qRT-PCR was conducted using the TaqPro Universal SYBR qPCR Master Mix (Vazyme, China). Each group was tested three times. Sangon Biotech (Shanghai, China) synthesized all primers and the sequences used were as follows: RUNX2-forward: GTGATAAATTCAGAAGGGAGG; RUNX2-reverse: CTTTTGCTAATGCTTCGTGT; COL1A1-forward: TCCAACGAGATCGAGATCC; COL1A1-reverse: AAGCCGAATTCCTGGTCT; OPN-forward: TTCCAAGTAAGTCCAACGAAAG; OPN-reverse: GTGACCAGTTCATCAGATTCAT; OSX-forward: GGGGCTGGATAAGCATCCC; OSX-reverse: CACAAAGAAGCCGTACTCTGT; CD31-forward: GACGTGCTCTTTTACAACATCTC; CD31-reverse: CCTCACGATCCCACCTTGG; VEGF-forward: AGGAGGAGGGCAGAATCATCA; VEGF-reverse: CTCGATTGGATGGCAGTAGCT; SOX9-forward: GGCGGAGGAAGTCGGTGAAGAA; SOX9-reverse: GCTCATGCCGGAGGAGGAGTGT; COL2A1-forward: GTCTGTGACACTGGGACTGT; COL2A1-reverse: TCTCCGAAGGGGATCTCAGG; AP2-forward: TGGTTGATTTTCCATCCCAT; AP2-reverse: TACTGGGCCAGGAATTTGAT; PPAR-γ2-forward: GCTGTTATG GGTGAAACTCTG; PPAR-γ2-reverse: ATAAGGTGGAGATGCAGGTTC; IL-1-forward: GGACAGGATATGGAGCAACAAGTGG; IL-1-reverse: TCATCTTTCAACACGCAGGACAGG; IL-6-forward: GCCTTCGGTCCAGTTGCCTTC; IL-6-reverse:GTTCTGAAGAGGTGAGTGGCTGTC; TNF-α-forward: CCTCATCTACTCCCAGGTCCTCTTC; TNF-α-reverse: TCTGGTAGGAGACGGCGATGC; IL-10-forward: GGCAACAGCATTATGACCCA; IL-10-reverse: TGAGATCGATGATGGCACTCC; TGF-β-forward: AAATTGAGGGCTTTCGCCTTA; TGF-β-reverse: TGAGATCGATGATGGCACTCC; GAPDH-forward: TGACGCTGGGGCTGGCATTG; GAPDH-reverse: GGCTGGTGGTCCAGGGGTCT.

### 2.13 Cytokine profiling of spheroid-secreted factors via protein microarray

To assess the secretory profiles of SVF- and ADSC-derived organoids, the spheroids that were cultured under GM or OM conditions for 14 days were transferred to serum-free medium and cultured for an additional 24 h. Subsequently, the conditioned medium was collected and analyzed using a customized RayBio® 120-target protein microarray to detect inflammatory cytokines, chemokines, GFs, and other secreted molecules. Each group was analyzed four times (n = 4). Finally, fluorescent signals were acquired using a laser scanner (InnoScan 300, 532 nm excitation), and the data were analyzed using QAH-CAA-2000 software to generate expression profiles for the secreted factors.

### 2.14 Inflammatory stimulation assay and multiplex IF Co-localization

First, P0 ADSCs and SVF cells were seeded at a density of 3 × 10^5^ cells/well in ultra-low attachment 96-well U-bottom plates. On day 3 post-formation, the spheroids were stimulated with a medium containing 250 ng/mL Lipopolysaccharide (LPS) for 1, 3, 7 consecutive days, creating the SVF + LPS and ADSC + LPS groups. The SVF and ADSC groups, which were cultured without LPS, served as controls. After LPS stimulation, two assays were performed. First, for multiplex IF co-localization, the spheroids were collected, fixed, dehydrated, embedded in OCT compound, and cryosectioned into 5-μm-thick slices. The samples were then assessed through multiplex IF co-localization for the expression levels of inflammation-related proteins, including Interleukin 1 (IL-1; Abcam ab2105, USA), IL-10 (Abcam ab133575, USA), CD206 (Abcam ab64693, USA), CD86 (Abcam ab239075, USA), and CD68 (Abcam ab955, USA), as previously described. Subsequently, cell nuclei were counterstained with DAPI, images were captured using a multispectral whole-slide imaging system (Akoya, USA). Since ADSC spheroids contained very few leukocytes, only IL-1, IL-10, and DAPI staining was performed for the ADSC group. Quantitative analysis was performed using ImageJ software. Each group was analyzed three times. Second, for qRT-PCR analysis, total RNA was first extracted from spheroids and reverse-transcribed as previously described. Following that, qRT-PCR was conducted to assess the mRNA expression levels of pro-inflammatory (IL-1, IL-6, TNF-α) and anti-inflammatory (IL-10 and TGF-β) factors, with GAPDH serving as the internal control. RNA was extracted from SVF + LPS and ADSC + LPS groups stimulated with LPS for 1, 3, and 7 days, as well as their respective control groups (SVF and ADSC spheroids), and then reverse-transcribed as described in Section 2.12. Each group was analyzed three times.

### 2.15 Proteomic analysis

The four groups [SVF GM, SVF OM, ADSC GM, and ADSC OM (n = 4 per group)] were subjected to proteomic analysis after 14 days of spheroid culture to explore the functional differences between SVF-derived organoids and ADSC spheroids under GM and OM conditions. After collection, all spheroids were immediately snap-frozen and stored at −80 °C. To eliminate erythrocytes, the SVF samples were first treated with RBC lysis buffer. Proteins were then extracted using 8 M urea lysis buffer containing a 1 % protease inhibitor cocktail. Subsequently, the samples were ultrasonicated to achieve cell lysis, and protein concentration was determined using the Bicinchoninic Acid (BCA) assay. Before enzymatic digestion, the proteins were precipitated and washed with pre-chilled acetone. Pellets were then collected and resuspended in 200 mM Triethylammonium Bicarbonate (TEAB) and digested overnight with trypsin at a 1:50 (enzyme: protein, w/w) ratio. Finally, reduction and alkylation were performed sequentially using 5 mM Dithiothreitol (DTT) at 56 °C for 30 min and 11 mM Iodoacetamide (IAA) at Room Temperature (RT) in the dark for 15 min, respectively.

On the other hand, peptides were separated on a NanoElute Ultra-High-Performance Liquid Chromatography (UHPLC) system and analyzed using a timsTOF Pro mass spectrometer in a data-independent acquisition mode with parallel accumulation-serial fragmentation (dia-PASEF). The MS1 scans were acquired over an *m/z* range of 300–1500 and were followed by 20 PASEF MS/MS scans over an *m/z* range of 400–8500. Raw data were processed using the Spectronaut software (version 18), with searches conducted against the Homo_sapiens_9606_SP_20231220 database (20,429 entries including reverse decoys). Trypsin/P was the specified digestion enzyme, allowing up to two missed cleavages. Carbamidomethylation of cysteine was set as a fixed modification, while variable modifications included N-terminal acetylation and methionine oxidation. The False Discovery Rate (FDR) was controlled at <1 % at both the peptide and protein levels.

Functional enrichment analysis of Differentially Expressed Proteins (DEPs) was performed using the total set of identified proteins as the background. Enrichment significance was determined using Fisher's exact test, with a fold enrichment >1.5 and P < 0.05 considered significant. Furthermore, P-values were transformed to -Log_10_(P) and hierarchically clustered using Euclidean distance and average linkage. Heatmaps were generated using the R package ComplexHeatmap. Protein–Protein Interaction (PPI) networks were also constructed using the STRING database, based on a confidence score threshold >0.7. Only intra-group interactions were retained, and visualization was performed using the R visNetwork package.

### 2.16 Nude mouse femoral fracture model and spheroid-based cell therapy

Herein, a femoral fracture model was established in nude mice to evaluate the four spheroid groups' bone regenerative efficacy. First, 16 six-week-old BALB/c nude mice were anesthetized with sodium pentobarbital. A longitudinal skin incision was then made along the medial aspect of the right thigh, before inserting an intramedullary pin along the axis of the femur. The pin's tail end was trimmed, and a transverse mid-diaphyseal femoral fracture was created using ophthalmic scissors. Subsequently, three spheroids from the corresponding experimental group were gently placed into the fracture gap using ophthalmic forceps. Due to their inherent adhesiveness and elasticity, the spheroids were able to remain largely in place, and muscle closure was performed to provide additional physical fixation. The control group (n = 4) received no spheroid implantation, while the sham group (n = 4) underwent pin insertion without fracture. All animals were sutured postoperatively and kept under standard conditions. The Animal Welfare and Ethics Committee of Zhongyan Zichuang (Beijing) Biotechnology Co., Ltd. approved all experimental procedures (Approval No. ZYZC202404019S).

### 2.17 Gross observation, radiography, and micro-computed tomography (Micro-CT)

At 4 weeks post-surgery, the right femurs were harvested, the soft tissues were removed, and the bones were fixed in 4 % PFA. Gross morphology was assessed and documented using a stereomicroscope (Nikon DS-Ri2, Japan). Radiographic imaging was conducted using the Faxitron MX-20 X-ray system (Faxitron, USA) to monitor fracture healing. High-resolution micro-CT was conducted using a SCANCO MicroCT μCT100 scanner (Scanco Medical, Switzerland) at an isotropic voxel size of 5 μm, with the scanning parameters set at 70 kV and 130 μA, using a 0.5 mm aluminum filter. Images were analyzed using CTAn software (Version 1.18), with quantitative parameters including Bone Volume (BV), Total Volume (TV), Bone Volume Fraction (BV/TV), and Bone Mineral Density (BMD). Each group was analyzed four times (n = 4).

### 2.18 Histological and immunohistochemical staining of fracture tissue

Bone specimens were fixed in 4 % PFA and decalcified in 15 % Ethylenediaminetetraacetic Acid (EDTA) for two weeks. After paraffin embedding, coronal sections (5 μm thick) were prepared, with several consecutive sections obtained from the central fracture region. Subsequently, H&E staining was performed to assess overall tissue structure at the fracture site. Masson's trichrome staining was further employed to evaluate newly formed bone and collagen distribution. Cartilage formation was visualized using Safranin O staining, while Tartrate-Resistant Acid Phosphatase (TRAP) staining was employed to detect osteoclast activity. On the other hand, whole-slide imaging was performed using a panoramic confocal digital slide scanner (DynaMax Biotech, China). Each group included four biological replicates (n = 4).

For immunofluorescence staining, paraffin sections were deparaffinized, rehydrated, and subjected to antigen retrieval, followed by incubation with antibodies against human osteocalcin (hOCN) (Abcam ab198228, USA) and mouse osteocalcin (mOCN) (Thermo Fisher Scientific PA5-78870, USA). Nuclei were counterstained with DAPI. Whole-slide images were captured using a multispectral imaging system (Akoya, USA), and quantitative analysis was performed using ImageJ software. Each group included four biological replicates (n = 4).

### 2.19 Statistical analysis

All statistical analyses were conducted using GraphPad Prism version 10.0, with all data presented as mean ± standard deviation (mean ± SD). Inter-group comparisons were performed using an independent-samples *t*-test, while multiple group comparisons were conducted using one-way or two-way Analysis of Variance (ANOVA), followed by Tukey's post hoc test. Results with P < 0.05 were considered statistically significant.

## 3 Results

### 3.1 Construction of SVF-derived organoids and assessment of their viability stability

**Fig. 2:**
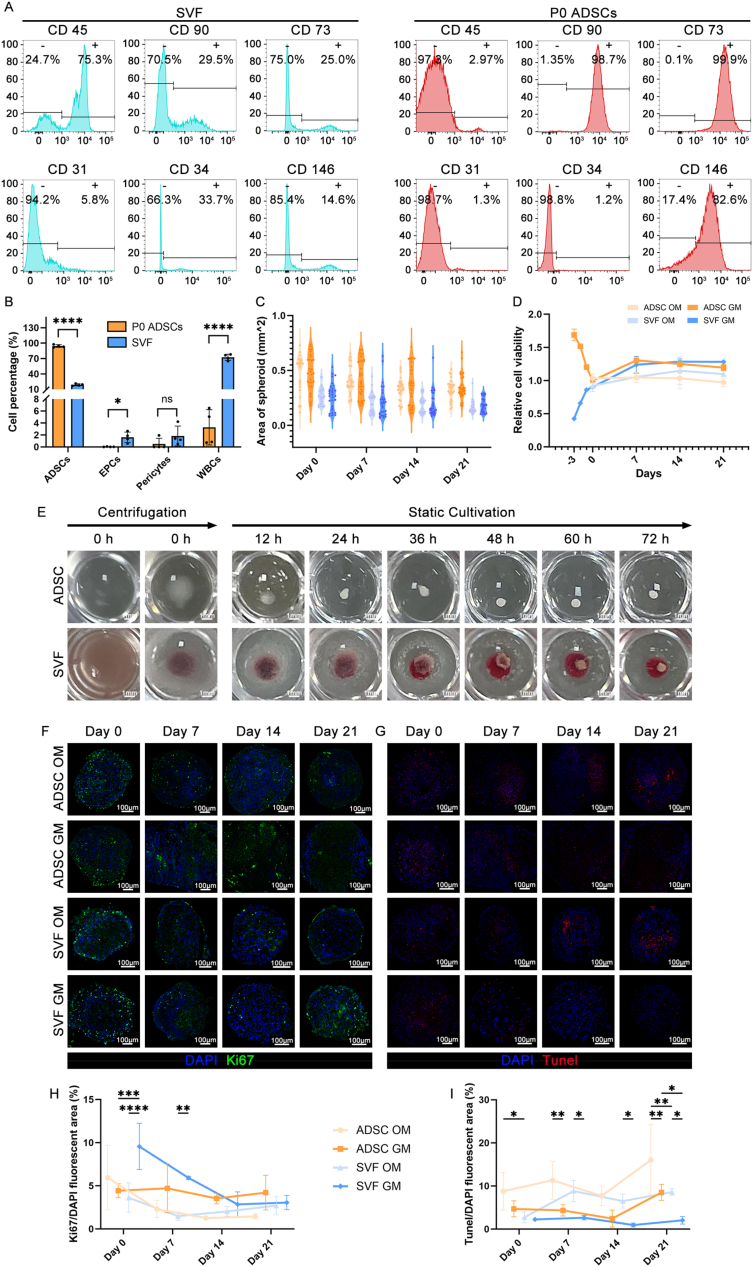
Profiling of SVF and P0 ADSCs via Flow cytometry; macroscopic analysis of spheroid self-assembly; and assessment of spheroid size, cell viability, proliferation, and apoptosis under different conditions. a) Representative flow cytometry plots for freshly isolated SVF and P0 ADSCs; b) Quantification of cell subpopulations (ADSCs, EPCs, pericytes, WBCs) in SVF and P0 ADSCs based on flow cytometry results; c) Alterations in spheroid area over time for SVF and P0 ADSCs cultured in growth medium (GM) and osteogenic medium (OM); d) Cell viability dynamics during the culture period in the indicated groups as determined by the Presto Blue assay; e) Representative macroscopic images showing the spheroid formation process in SVF and P0 ADSCs; f, h) Immunofluorescence staining for Ki67 to analyze cell proliferation and quantify the Ki67-positive cells at the indicated time points; g, i) TUNEL staining to evaluate apoptotic cells and quantification of TUNEL-positive cells in spheroids at different time points.

Herein, freshly isolated human SVF cells and P0 ADSCs, obtained via 2D culture expansion of the same donor's SVF, were characterized using flow cytometry to identify and quantify distinct cell populations and the proportions of various markers (Fig. 2A, Supplementary Fig. S1A and B). Based on six representative markers**,** compared to SVF (18.76 ± 2.06 %), P0 ADSCs exhibited a significantly enriched population of ADSCs (CD45^-^/CD73^+^/CD90^+^; 94.35 ± 2.616 %) (Fig. 2B). Conversely, the population of EPCs (CD45^-^/CD31^+^/CD34^+^) was markedly lower in ADSCs (0.03 ± 0.04 %) than in SVF (1.61 ± 0.88 %). Furthermore, both ADSCs (0.49 ± 0.97 %) and SVF (1.84 ± 1.64 %) exhibited comparable levels of pericytes (CD45^-^/CD73^-^/CD90^-^/CD146^+^). Meanwhile, the leukocyte population (CD45^+^) was significantly lower in ADSCs (3.28 ± 2.94 %) than in SVF (72.40 ± 6.05 %). Given the substantial proportion of leukocytes in SVF and previous reports indicating a high macrophage content in SVF [32], we further performed flow cytometric analysis to identify macrophages and their subtypes within SVF. The results showed that M1 macrophages (CD86^+^/CD206^-^) accounted for 1.41 ± 0.21 %, M2 macrophages (CD86^-^/CD206^+^) for 0.24 ± 0.29 %, while double-positive macrophages (CD86^+^/CD206^+^) made up a significantly higher proportion at 15.28 ± 1.41 % **(**Supplementary Fig. S1C and D**)**. Moreover, the monolayer expansion process led to a near-complete erythrocyte loss. These findings indicate that following adherent culture, ADSCs were the predominant cell type in SVF cultures.

The SVF and P0 ADSCs (hereafter referred to as ADSCs) were subsequently compared in terms of spheroid formation using a centrifugation-based aggregation method in ultra-adhesive 96-well plates (Fig. 2E). Whereas ADSCs rapidly formed spheroids within 12 h, the SVF initially formed a peripheral white membrane atop a dense layer of RBCs, followed by a slower aggregation process that completed by 72 h. Ultimately, the two types of spheroids exhibited similar morphology and were used in all subsequent experiments, with cells from the same donor employed as matched controls.

The diameters of SVF and ADSC spheroids formed under GM and OM conditions were also compared (Fig. 2C). Although the SVF and ADSCs (excluding RBCs) had a similar total initial seeding number (300,000 cells/well), ADSC spheroids were consistently larger. Nonetheless, both spheroid types gradually decreased in size over time, with both groups exhibiting smaller diameters in OM conditions compared to GM conditions. This phenomenon could be attributed to ADSCs’ higher proliferative capacity retained immediately after 2D culture, with SVF cells potentially requiring an adaptation period following transition from the *in vivo* environment to *in vitro* conditions.

To test the aforementioned hypothesis, we assessed cell viability over time using Presto Blue assays (Fig. 2D). The most dramatic changes occurred between days −3 and 0, with a significant decline and increase in viability observed in ADSCs and SVF, respectively. Nonetheless, both groups exhibited comparable viability by day 0 (immediately after spheroid formation). Thereafter, cell viability initially increased under both GM and OM conditions, and then decreased. Notably, GM-cultured SVF spheroids showed no decline by day 21, with OM-cultured spheroids exhibiting a lower viability.

To further assess proliferation and apoptosis, we performed Ki67 (Fig. 2F–H) and TUNEL staining (Fig. 2G–I). According to the results, all groups showed a time-dependent decrease in proliferative cells and an increase in apoptotic cells. Overall, compared to ADSC spheroids, SVF spheroids exhibited higher and lower percentages of Ki67-positive and TUNEL-positive cells, respectively. Furthermore, compared to OM-cultured spheroids, those in GM consistently showed higher proliferation and lower apoptosis levels.

### 3.2 SVF-derived organoids underwent spontaneous *in vitro* maturation and developed complex structures

**Fig. 3:**
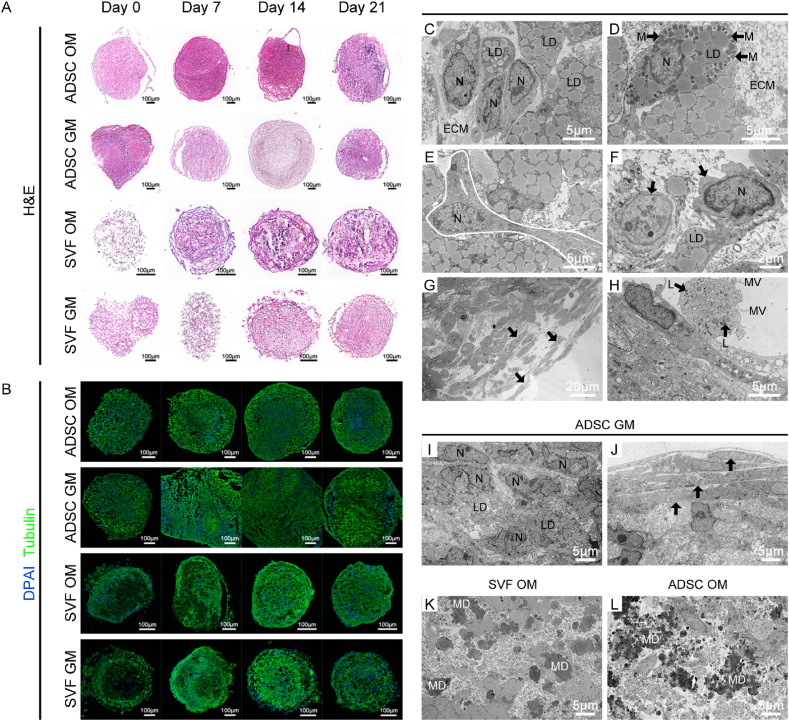
Morphological staining and transmission electron microscopy (TEM) analysis of spheroids derived from SVF-GM, SVF-OM, ADSC-GM, and ADSC-OM groups. a) Hematoxylin and eosin (H&E) staining of spheroids from the four groups (SVF-GM, SVF-OM, ADSC-GM, and ADSC-OM) at days 0, 7, 14, and 21, illustrating the dynamic structural changes during culture; b) Immunofluorescence staining for α-tubulin (cytoskeleton) and DAPI (nuclei), showing cellular morphology and spatial arrangement within spheroids; c–h) Transmission electron microscopy (TEM) images of SVF-GM spheroids on day 14, highlighting ultrastructural features of various cell types and extracellular matrix components; i–j) TEM images of ADSC-GM spheroids on day 14; k) TEM image of SVF-OM spheroids on day 14; l) TEM image of ADSC-OM spheroids on day 14. N, nucleus; M, mitochondria; LD, lipid droplet; ECM, extracellular matrix; MV, microvilli; L, lysosome; MD, mineral deposition.

To further explore compositional and structural differences between SVF-derived organoids and ADSC spheroids, we performed H&E staining and IF staining for tubulin (Fig. 3A and B). According to the histological images, SVF-derived organoids exhibited a more organized cellular arrangement, richer ECM, and looser intercellular spacing compared to ADSC spheroids, which appeared as compact cellular aggregates. The cytoskeletal morphology in SVF organoids was also more natural and physiologically relevant. On the other hand, H&E images revealed that OM-cultured spheroids had disordered central structures, indicating a potentially unfavorable microenvironment for cell survival.

We then subjected day 14 spheroids to TEM to assess the ultrastructural attributes of SVF-derived organoids and confirm their heterotypic cellular composition—distinct from the homogeneous expansion of ADSCs. In GM-cultured SVF organoids, the spheroid core comprised interconnected ADSCs, which contained numerous small lipid droplets (Fig. 3C) and were surrounded by abundant ECM. Mitochondria-enriched cells were also observed (Fig. 3D), implying high metabolic activity. Additionally, ADSCs in the spheroid center occasionally exhibited elongated, endothelial-like cells, as indicated by the white line in (Fig. 3E), indicating the presence of EPCs, which established extensive intercellular junctions. Some non-lipid-containing cells with an unclear identity were also observed, as indicated by the arrows in (Fig. 3F).

Several layers of elongated cells containing lipid droplets, which could be stretched ADSCs, were also observed at the periphery of SVF organoids, as indicated by the arrows in (Fig. 3G). These cells distinctly contrasted with the irregularly shaped ADSCs in the core and appeared to form a barrier layer, potentially regulating substance exchange and creating a more favorable microenvironment for the internal cells. Moreover, cells with abundant phagolysosomes and microvilli, possibly macrophages, adhered to the outermost ADSCs (Fig. 3H).

Conversely, TEM of ADSC-GM spheroids showed densely packed ADSCs with tight intercellular contacts (Fig. 3I). Furthermore, the outer layers comprised several compact, elongated ADSCs, as indicated by the arrows in (Fig. 3J). Moreover, TEM analysis of SVF-OM and ADSC-OM spheroids (Fig. 3K and L) revealed substantial mineral deposition in both groups, with SVF spheroids exhibiting larger particulate deposits.

**Fig. 4:**
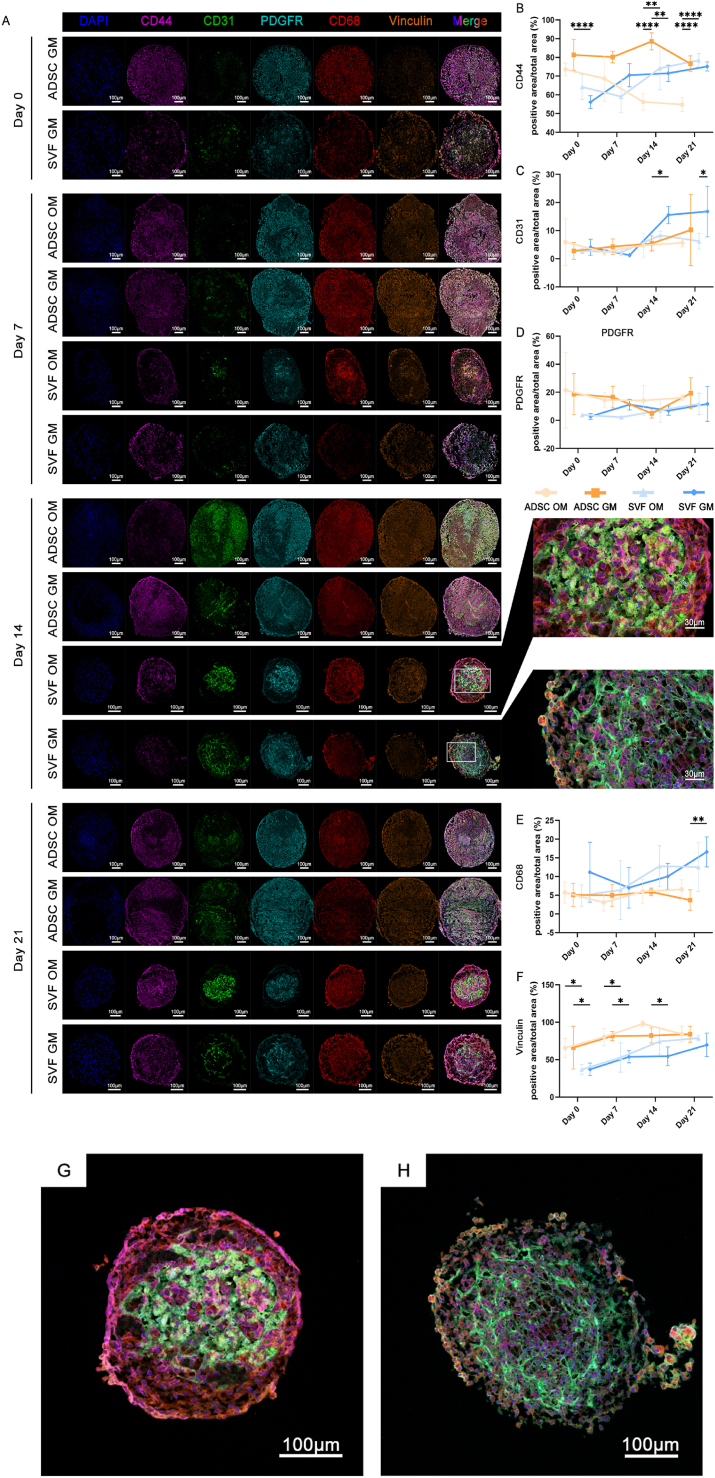
Multicolor immunofluorescence co-localization and quantitative analysis of spheroids from SVF-GM, SVF-OM, ADSC-GM, and ADSC-OM groups at the indicated time points, demonstrating the cellular composition and spatial organization. a) Representative multicolor immunofluorescence images of spheroids in the indicated four groups at days 0, 7, 14, and 21, showing nuclei (DAPI), adipose-derived mesenchymal stem cells (CD44, purple), endothelial cells (CD31, green), pericytes (PDGFR, cyan), M0 macrophages (CD68, red), cell adhesion protein (Vinculin, orange), and merged images (Merge), showing the spatial distribution of various cell populations within the spheroids; b–f) Quantitative analysis of the positive area ratios for CD44, CD31, PDGFR, CD68, and Vinculin staining, comparing the temporal and treatment-dependent changes in cellular composition within spheroids; g–h) Representative high-magnification merged images of SVF-GM and SVF-OM spheroids on day 14, highlighting differences in cellular arrangement and structure.

We further performed multicolor IF co-localization staining to characterize the cellular composition and spatial organization of SVF spheroids (Fig. 4A). At days 0 and 7 post-aggregation, SVF spheroids exhibited cellular heterogeneity but without an organized spatial structure. At day 14, a more mature architecture had developed, with abundant tubular structures of EPCs (CD31, green) forming an interwoven network. At day 14, the CD31-positive area in SVF-GM spheroids was significantly larger than that in ADSC-GM spheroids. Moreover, spheroids cultured under GM conditions exhibited higher CD31 expression than those in OM groups, although statistically significant differences were only observed between SVF-GM and SVF-OM groups at day 21 (Fig. 4C). Due to tight cell-cell contacts within spheroids, we quantified the fluorescent-positive area rather than cell counts.

Pericytes (PDGFR, cyan) were arranged in parallel with ECs without overlapping and showed similar abundance (Fig. 4D). Furthermore, ADSCs (CD44, purple) were distributed throughout the spheroid, representing the predominant cell type and overall exhibited a significantly higher proportion in ADSC spheroids than in SVF organoids (Fig. 4B). Additionally, macrophages (CD68, red) were primarily localized to the spheroid periphery, with significantly higher proportions in SVF spheroids than in ADSC spheroids, although a statistically significant difference was only observed between SVF-GM and ADSC-GM groups at day 21 (Fig. 4E). Moreover, the expression of the cell-cell adhesion protein vinculin (orange) was significantly higher in ADSC spheroids than in SVF organoids (Fig. 4F), potentially due to a higher overall cell density in ADSC spheroids.

We further assessed merged fluorescence images (Fig. 4G and H), revealing that SVF-GM spheroids exhibited a loosely organized, web-like cellular architecture, whereas OM-cultured SVF spheroids showed more aggregated, cluster-like arrangements of endothelial and pericyte populations. These structural changes suggest a disruption of capillary-like networks under osteogenic conditions, albeit without significant alterations in marker expression levels. Based on these insights, we hypothesized that the OM culture condition may suppress the integrated development of SVF-derived organoids.

The self-organizing capacity of SVF spheroids may originate from the intrinsic cellular heterogeneity and intercellular interactions within the SVF population. In particular, the crosstalk between endothelial cells and pericytes is thought to play a key role in vascular-like structure formation [33], while macrophage-derived chemokines and cytokines may contribute to multicellular spatial remodeling [34]. Moreover, dynamic regulation of cell–cell adhesion molecules and extracellular matrix components likely facilitates the structural self-organization of the spheroids.

Overall, SVF-derived organoids require ⁓14 days post-aggregation to mature and form complex, organized structures. Additionally, osteogenic induction could interfere with organoid structural integrity, while ADSC spheroids might lack such architectural complexity despite the presence of minor non-ADSC populations in P0 ADSCs.

### 3.3 SVF-derived organoids exhibited diverse biological functions

**Fig. 5:**
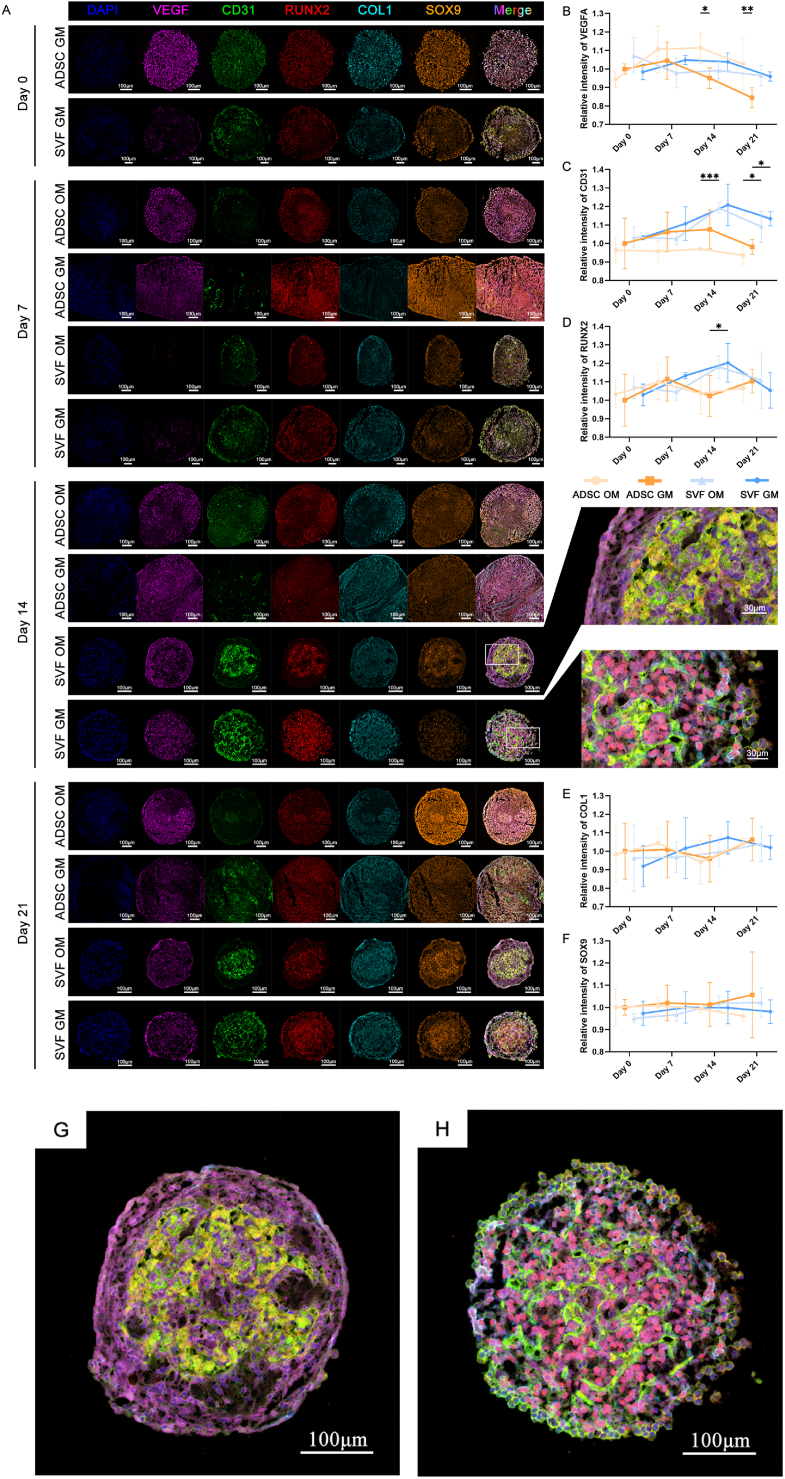
Representative multicolor immunofluorescence images showing co-localization and quantitative analysis of osteogenic, angiogenic, and chondrogenic markers in SVF-GM, SVF-OM, ADSC-GM, and ADSC-OM spheroids across different time points. a) Representative multicolor immunofluorescence images of spheroids in the indicated four groups at days 0, 7, 14, and 21 of culture, showing nuclei (DAPI), vascular endothelial growth factor (VEGF), endothelial marker CD31, early osteogenic marker RUNX2, mid-to-late osteogenic marker COL1, chondrogenic marker SOX9, and merged images (Merge), urevealing the spatial distribution of lineage-specific markers during differentiation; b–f) Quantitative analysis of average fluorescence intensity for VEGF, CD31, RUNX2, COL1, and SOX9, comparing the expression levels across different groups and time points; g–h) High-magnification merged images of SVF-GM and SVF-OM spheroids at day 14 highlighting the structural differences in marker localization.

Given the well-established multilineage differentiation potential of MSCs, we sought to further establish whether SVF-derived spheroids, with their more complex cellular composition and structural organization, exhibited enhanced biological functions. We also evaluated the effects of different culture conditions on these functions—particularly the osteogenic, chondrogenic, and angiogenic capabilities of SVF-derived organoids (Fig. 5A). There were notable temporal differences in both the expression levels and spatial localization of lineage-specific biomarkers, with functional marker expression generally peaking at day 14.

Regarding angiogenesis, the expression levels of Vascular Endothelial Cell (VEC) markers—CD31 (green) and the Vascular Endothelial Growth Factor (VEGF, purple)—showed a generally increasing trend and later decreased over time. Moreover, SVF spheroids exhibited a higher expression of these markers than ADSC spheroids. Additionally, the GM outperformed the OM in promoting angiogenic marker expression, except for VEGF, which peaked in the ADSC-OM group (Fig. 5B and C).

For osteogenesis, SVF-GM spheroids showed a significantly higher expression of the early osteogenic Transcription Factor (TF) RUNX2 (red) than ADSC-GM spheroids at day 14, with other time points showing no significant differences. The SVF and ADSC groups also showed no significant difference in Type I Collagen (COL1, cyan) expression. Interestingly, no consistent superiority was observed between the GM and OM conditions regarding osteogenic marker expression (Fig. 5D and E).

As for chondrogenesis, no significant differences were observed in the expression of the chondrogenic TF SOX9 across groups or time points (Fig. 5F)—a phenomenon attributable to the absence of specific chondrogenic induction factors in the culture medium.

Spatial analysis of marker localization (Fig. 5G and H) revealed that angiogenic markers were predominantly expressed within the organoid core, forming a web-like vascular network that presumably facilitated nutrient and oxygen transport throughout the spheroid. Notably, VEGF was widely distributed across the entire SVF spheroid, potentially supporting vascular structure development and maintenance. Furthermore, RUNX2 expression was primarily localized within the spheroid core, exhibiting strong positive signals in the interstitial spaces between ECs, but a weak expression at the periphery. These findings collectively suggest that EC-MSC interactions may promote osteogenic differentiation. Furthermore, the peripheral elongated MSCs and immune cells provided structural support that might have created a microenvironment conducive to core cell survival and differentiation. Moreover, SOX9 expression exhibited a distribution pattern comparable to that of RUNX2 but at lower levels—potentially due to the lack of chondrogenic inducers in the culture conditions.

Necrotic tissue could lead to significant non-specific fluorescence staining, particularly in ADSC spheroids at days 14 and 21. In such cases, the spheroid core often lacked nuclear staining, indicating nutrient deprivation- and hypoxia-induced cell death. Nevertheless, considerable non-specific fluorescence signals from labeled markers remained detectable in these regions. Notably, we mainly selected areas with intact nuclear staining for quantitative analysis; however, such non-specific backgrounds could have affected the accuracy of fluorescence-based quantification.

**Fig. 6:**
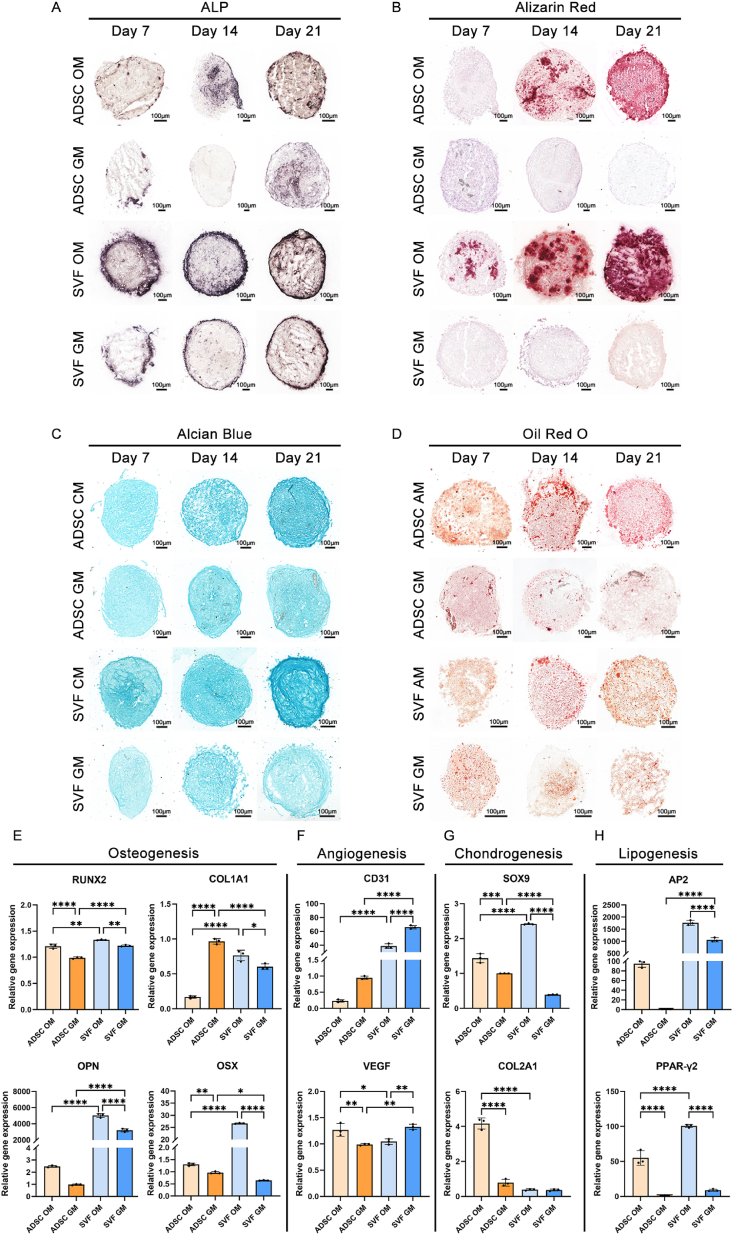
Differential staining and gene expression analysis of SVF-derived organoids and ADSC spheroids following osteogenic, chondrogenic, and adipogenic induction. a–b) Alkaline phosphatase (ALP) staining and Alizarin Red S staining of SVF-GM, SVF-OM, ADSC-GM, and ADSC-OM spheroids at days 7, 14, and 21, were performed to examine the osteogenic differentiation and mineralization levels; c) Representative Alcian Blue staining images showing SVF-GM, SVF-CM, ADSC-GM, and ADSC-CM spheroids at days 7, 14, and 21, evaluating chondrogenic matrix formation following chondrogenic induction; d) Oil Red O staining of SVF-GM, SVF-AM, ADSC-GM, and ADSC-AM spheroids at days 7, 14, and 21, assessing lipid droplet formation and adipogenic differentiation potential; e–h) Quantitative RT-PCR analysis was performed on SVF-GM, SVF-OM, ADSC-GM, and ADSC-OM spheroids at day 14 to assess the expression of functional lineage-related genes, including: e) Osteogenic markers: RUNX2, COL1A1, OPN, and OSX; f) Angiogenic markers: CD31 and VEGF; g) Chondrogenic markers: SOX9 and COL2A1; h) Adipogenic markers: AP2 and PPAR-γ2.

To further confirm the multifaceted biological functionality of SVF-derived organoids, we conducted several histochemical staining procedures—including ALP (Fig. 6A), ARS (Fig. 6B), ABS (Fig. 6C), and OROS (Fig. 6D)—at various time points under different culture conditions. We also conducted qPCR analysis at day 14 post-aggregation in spheroids from four groups: SVF-GM, SVF-OM, ADSC-GM, and ADSC-OM.

According to the ALP staining results, the SVF-OM group exhibited a significantly stronger expression than the ADSC-OM group. Interestingly, despite the absence of central necrosis, ALP expression in SVF organoids was predominantly localized to the spheroid periphery. Notably, even without osteogenic induction, the SVF-GM group exhibited a detectable level of ALP expression. Although lower than that observed in the SVF-OM group, it was still higher than that in the ADSC-GM, indicating the superior intrinsic osteogenic potential of SVF-derived organoids.

On the other hand, ARS revealed that mineral deposition became evident in the ADSC-OM group only after 14 days of induction, whereas small amounts of mineralization were observed in the SVF-OM group from as early as day 7. By day 21, mineral deposition in the SVF-OM group was markedly greater than that in the ADSC-OM group. Moreover, the non-induced groups showed no mineralization, confirming the enhanced osteogenic differentiation capacity of SVF organoids.

In ABS, which revealed chondrogenic matrix formation following chondrogenic induction, the SVF-CM group initiated cartilage matrix deposition earlier and more robustly than the ADSC-CM group, highlighting SVF organoids’ superior chondrogenic differentiation potential.

During adipogenic induction, both SVF and ADSC spheroids formed large lipid vacuoles by day 14, which became more prominent by day 21 and were primarily distributed at the spheroid periphery. Additionally, OROS revealed small lipid droplets in the non-induced GM groups (SVF-GM and ADSC-GM)—a phenomenon consistent with our earlier TEM observations, which revealed numerous small lipid droplets in ADSCs. Notably, these droplets decreased progressively with prolonged culture.

To further investigate the molecular basis of differentiation, day 14 spheroids from the SVF and ADSC groups that were cultured under both GM and OM conditions were subjected to qPCR analysis. All gene expression levels were normalized to the ADSC-GM group. For osteogenic markers (Fig. 6E), except for COL1α1, which was unexpectedly lower in the ADSC-OM group, all other expressions were generally higher under OM conditions compared to GM conditions. Furthermore, the ADSC-GM group showed a higher COL1α1 and OSX expression than the SVF-GM group, whereas RUNX2 and OPN were more highly expressed in the SVF-GM group, with OPN exhibiting a particularly significant upregulation, thereby suggesting a potentially distinct osteogenic regulatory mechanism in SVF organoids. Given that OPN is involved not only in osteogenesis but also in inflammation regulation and cell migration, its marked upregulation—together with the multicellular composition of SVF—may indicate an “immune-regulatory” osteogenic state. This could reflect a multicellular cooperative mechanism that more closely mimics the natural bone repair microenvironment [35].

For angiogenesis-related genes (Fig. 6F), the SVF exhibited a significantly higher CD31 and VEGF expression than ADSCs. Furthermore, osteogenic induction significantly downregulated CD31 and VEGF, although VEGF expression was unexpectedly elevated in the ADSC-OM group.

Regarding chondrogenic genes (Fig. 6G), although the expression of chondrogenic markers is rarely assessed under osteogenic induction conditions, our qPCR results revealed that both SOX9 and COL2α1 were upregulated under OM conditions compared to GM conditions. Notably, COL2α1 expression did not show a significant difference between SVF-OM and SVF-GM groups. These findings suggest that osteogenic induction may also promote the expression of chondrogenic markers in mesenchymal stem cell–derived spheroids, possibly indicating the activation of chondrogenic pathways during osteogenic differentiation. This, in turn, implies a potential crosstalk or regulatory overlap between osteogenic and chondrogenic differentiation programs. Nevertheless, immunofluorescence staining for SOX9 showed no significant differences among groups (Fig. 5F).

Interestingly, the analysis of adipogenesis-related gene expression (Fig. 6H) revealed that osteogenic induction also significantly upregulated adipogenic genes such as AP2 and PPAR-γ in both SVF and ADSC spheroids, indicating that crosstalk or regulatory overlap may also exist between the osteogenic and adipogenic differentiation pathways.

### 3.4 SVF-derived organoids modulated immune responses and adapted to inflammatory microenvironments (IMEs)

Since secreted protein factors could crucially mediate the host response to implanted organoids, we explored the secretory profiles of SVF-derived organoids and ADSC spheroids cultured under GM and OM conditions for 14 days. Following the culture period, the spheroids were incubated in serum-free medium for 24 h. Conditioned media were then collected and analyzed. Subsequently, 120 commonly studied cytokines, chemokines, and GFs were profiled using a custom protein microarray (RayBio).

**Fig. 7:**
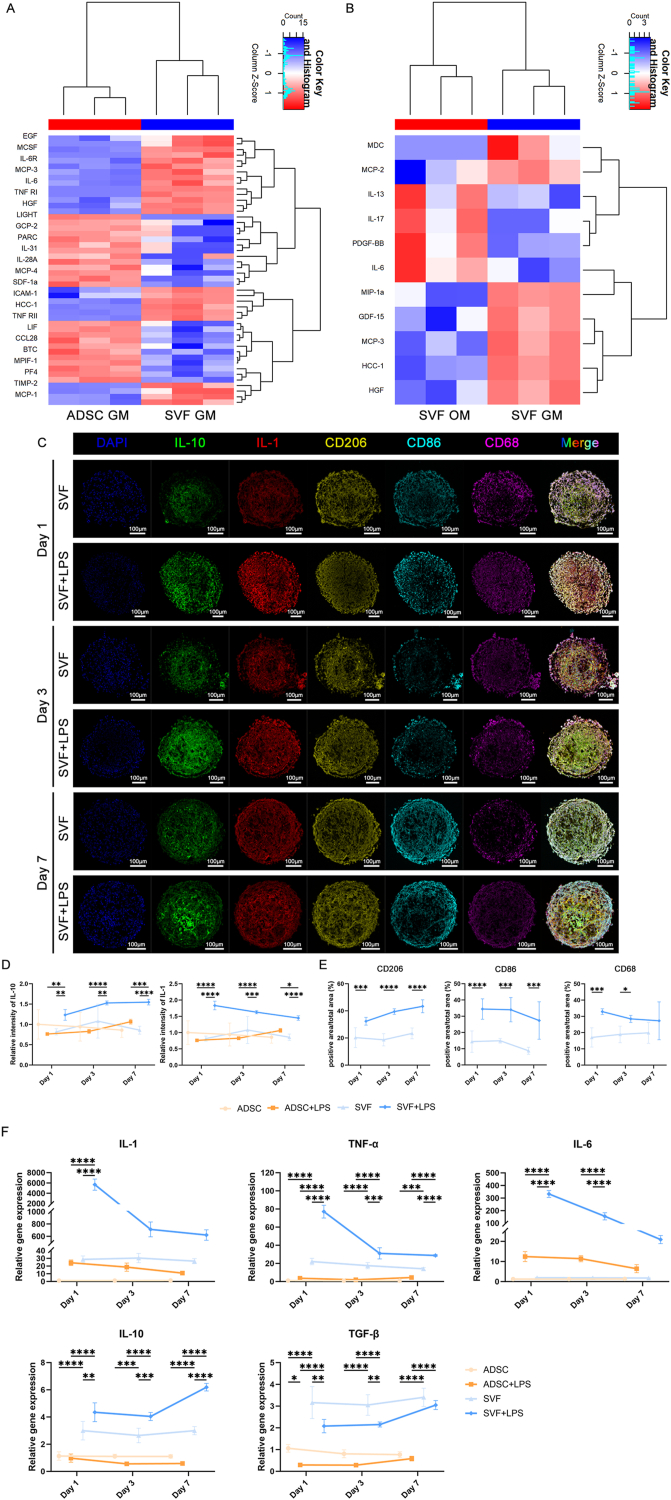
Profiling the differential protein secretion and inflammatory responses induced by SVF-derived organoids and ADSC spheroids following LPS stimulation. a–b) Heatmaps of protein array analysis comparing the secreted protein profiles in conditioned media collected at day 14: a) SVF-GM vs. ADSC-GM; b) SVF-GM vs. SVF-OM. A total of 120 soluble protein factors—including cytokines, chemokines, and growth factors—were analyzed, revealing distinct expression patterns between groups; c) Representative multicolor immunofluorescence staining images showing the co-localization staining of SVF Organoids after 1, 3, and7 days incubation with lipopolysaccharide (LPS), compared with their respective non-LPS controls. Markers such as, DAPI (nuclei), IL-10 (anti-inflammatory cytokine), IL-1 (pro-inflammatory cytokine), CD206 (M2 macrophage marker), CD86 (M1 macrophage marker), CD68 (M0 macrophage marker), and merged images (Merge); d) Quantification of the average fluorescence intensity for IL-10 and IL-1 in ADSC, ADSC + LPS, SVF, and SVF + LPS groups; e) Quantitative analysis of the proportion of positive staining area for CD206, CD86, and CD68 in SVF and SVF + LPS groups; f) qRT-PCR analysis of pro-inflammatory genes (IL-1, TNF-α, IL-6) and anti-inflammatory genes (IL-10, TGF-β) in SVF and ADSC spheroids following LPS stimulation for 1, 3, and 7 days, and in their respective non-stimulated controls.

Compared to that from ADSC-GM spheroids, the conditioned medium from SVF-GM spheroids showed higher levels of protein factors primarily involved in cell proliferation, chemotaxis, migration, inflammation, and immune regulation, such as EGF, M-CSF, MCP-3, ICAM-1, MCP-1, IL-6, and TNF-RI (Fig. 7A). Interestingly, the proteins downregulated between SVF-GM and ADSC-GM—including CCL28, MCP-4, and GCP-2—were also largely associated with inflammation and immune responses (Fig. 7A), implying an immune-modulatory capacity in SVF-derived organoids that might support immune homeostasis.

Fewer Differentially Expressed Proteins (DEPs) were observed between the SVF-GM and SVF-OM groups. Among those upregulated in SVF-GM, some, including MDC, MCP-2, HGF, and MIP-1α, were associated with immune cell regulation, inflammatory responses, and tissue repair (Fig. 7B). Similarly, the proteins downregulated in SVF-GM, including IL-17 and IL-6, were predominantly linked to immune and inflammatory regulation (Fig. 7B).

To verify these seemingly paradoxical findings, we exposed SVF organoids to LPS for 1, 3, and 7 days to simulate an IME, with ADSC spheroids serving as controls. Multicolor IF staining revealed that both SVF and ADSC spheroids exhibited an increased expression of the pro-inflammatory cytokine IL-1 and the anti-inflammatory cytokine IL-10 under LPS stimulation, with SVF organoids exhibiting a more pronounced response. Specifically, the SVF + LPS group showed significantly higher levels of both IL-1 and IL-10 compared to the ADSC + LPS group. Moreover, over time, IL-10 expression in SVF organoids displayed an upward trend, while IL-1 levels gradually declined (Fig. 7C and D; Supplementary Fig. S2).

We also found that IL-1 and the M1 macrophage marker CD86 (cyan) were predominantly expressed at the periphery of SVF organoids, whereas IL-10 and the M2 macrophage marker CD206 (yellow) were enriched at the core. In addition, compared to untreated SVF controls, the SVF + LPS group exhibited a significantly increased positive staining area for CD86^+^ M1 macrophages, which gradually decreased over time. In contrast, the positive area for CD206^+^ M2 macrophages also increased significantly and showed a progressive upward trend over time. Moreover, the proportion of M0 macrophages initially increased under LPS stimulation but gradually declined with prolonged exposure (Fig. 7C and E).

qPCR analysis further confirmed these findings (Fig. 7F). Compared to the untreated controls, both SVF organoids and ADSC spheroids exhibited overall upregulation of pro-inflammatory genes (IL-1, TNF-α, IL-6) and anti-inflammatory genes (IL-10, TGF-β) after LPS stimulation at days 1, 3, and 7, with SVF organoids consistently showing higher expression levels than ADSC spheroids across all time points. Notably, in the SVF + LPS group, pro-inflammatory cytokines (IL-1, TNF-α, and IL-6) showed a clear downward trend over time, while anti-inflammatory cytokines (IL-10 and TGF-β) displayed a progressive increase. In contrast, ADSC spheroids showed lower IL-10 and TGF-β expression in the LPS-treated group compared to controls. Although TGF-β expression in SVF organoids under LPS stimulation was slightly reduced at early time points, it still demonstrated an overall upward trend over time.

These findings collectively suggest that SVF-derived organoids exhibited a stronger immunomodulatory capacity than ADSC spheroids. Upon exposure to injury or infection, the peripheral cells of SVF organoids enhanced pro-inflammatory responses to rapidly control and eliminate pathogens or damaged tissue. Concurrently, the organoid core exhibited an upregulated anti-inflammatory response, preventing excessive inflammation and promoting tissue repair. This response pattern mirrors the *in vivo* immune regulation process, which is essentially a dynamic balance between pro-and anti-inflammatory signals. The coordinated action of various signaling molecules ensures that the immune system responds effectively to external threats while minimizing collateral damage to host tissues.

### 3.5 Proteomic profiling revealed differential protein expression between SVF organoids and ADSC spheroids under GM and OM conditions

**Fig. 8:**
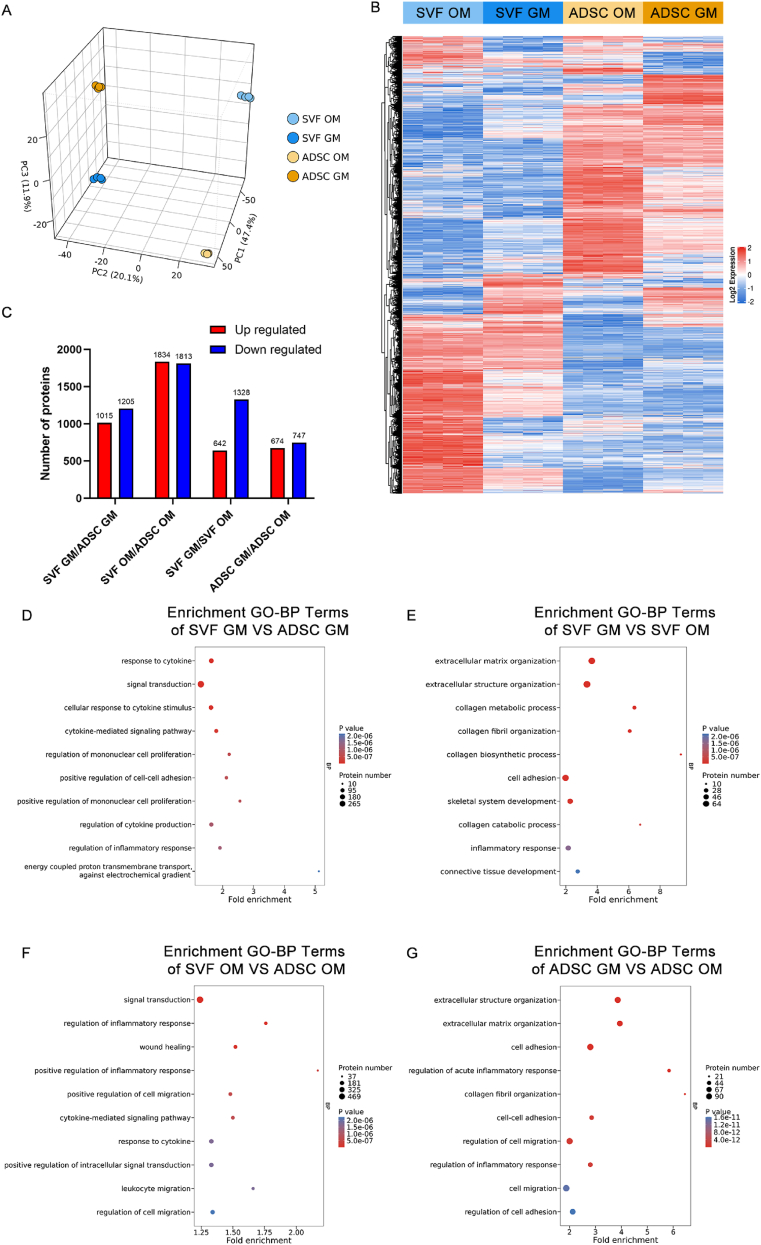
Proteomic profiling of SVF-GM, SVF-OM, ADSC-GM, and ADSC-OM spheroids. a) Principal component analysis (PCA) based on proteomic results from day 14 samples, displaying global differences in protein expression profiles across the four groups; b) Heatmap of differentially expressed proteins in the indicated four groups, demonstrating the influence of cell source and differentiation conditions on proteomic signatures; c) Quantification of the number of significantly upregulated and downregulated proteins in each pairwise group comparison; d–g) Gene Ontology Biological Process (GO-BP) enrichment analysis of upregulated differentially expressed proteins in the following comparisons, showing the top 10 enriched biological processes: d) SVF-GM vs. ADSC-GM; e) SVF-GM vs. SVF-OM; f) SVF-OM vs. ADSC-OM; g) ADSC-GM vs. ADSC-OM.

We performed a comprehensive proteomic analysis across the four groups to further elucidate the functional differences between SVF-derived organoids and ADSC spheroids under GM and OM conditions. Principal Component Analysis (PCA) revealed that SVF-GM samples from four individual donors exhibited tightly clustered and clearly distinct protein expression profiles relative to ADSC-GM samples. Upon osteogenic induction, both the SVF-OM and ADSC-OM groups exhibited noticeable shifts in protein expression patterns compared to their respective GM counterparts, even within the same donor (Fig. 8A).

Hierarchical clustering heatmaps further demonstrated a high similarity among biological replicates and a low inter-donor variability. Notably, SVF organoids and ADSC spheroids displayed distinct global protein expression profiles. Meanwhile, differences between the GM and OM conditions within the same cell type, although relatively smaller, were present (Fig. 8B). In addition, the widespread distribution of data points in the volcano plots highlights significant differences in protein expression patterns between spheroids derived from different cell sources and culture conditions, with several differentially expressed proteins identified that are related to immunomodulation (e.g., ITGB2, CD36, HLA-DRB5, HLA-C, ERAP2, CD53, LSP1, EPX), angiogenesis (e.g., F13A1, MMP1, PLG), and osteogenesis (e.g., COL1A1, COL12A1, COL14A1, EFEMP2, MGP, POSTN) **(**Supplementary Fig. S3A–D**)**.

We identified 7802 proteins across all groups. According to the differential expression analysis results, compared to ADSC-GM, SVF-GM showed 1015 and 1205 proteins with higher and lower expression levels, respectively. Similarly, SVF-OM exhibited 1834 and 1813 proteins with higher and lower expressions, respectively. In comparing the GM and OM conditions within the same cell type, relative to SVF-OM, SVF-GM showed 642 and 1328 proteins with higher and lower expressions, respectively. Meanwhile, compared to ADSC-OM, ADSC-GM had 674 and 747 proteins with higher and lower expressions, respectively (Fig. 8C). These findings indicate that the SVF and ADSCs exhibited markedly different proteomic landscapes, and that culture conditions (GM vs. OM) can significantly modulate protein expression patterns within each cell type.

To further explore the biological implications of these differences, Gene Ontology (GO) enrichment analysis was performed based on pairwise comparisons, focusing on the top 50 significantly enriched biological processes (Biological Process, BP) that were upregulated in intergroup comparisons **(**Supplementary Fig. S4–S7**)**. Compared to ADSC-GM, the upregulated proteins in SVF-GM were mainly enriched in GO terms related to cytokine signaling, inflammation regulation, and energy metabolism (Fig. 8D). Between SVF-GM and SVF-OM, the upregulated proteins in SVF-GM were enriched in terms such as ECM organization and collagen biosynthesis (Fig. 8E). Furthermore, compared to ADSC-OM, the upregulated proteins in SVF-OM showed enrichment in processes such as cytokine-mediated signaling, inflammatory response, and tissue repair (Fig. 8F). Conversely, between ADSC-GM and ADSC-OM, the upregulated proteins in ADSC-GM were primarily enriched in GO terms associated with ECM organization and cell migration (Fig. 8G).

These findings collectively suggest that SVF organoids exhibited enhanced capacities for cytokine signaling and immune regulation compared to ADSC spheroids. Additionally, compared to those under OM conditions, GM-cultured cells exhibited stronger functional enrichment in ECM production, cell proliferation, and migration. These results may provide a molecular basis for SVF organoids’ superior biological responsiveness and regenerative potential.

### 3.6 *In vivo* evaluation of SVF-derived organoids for fracture repair

**Fig. 9:**
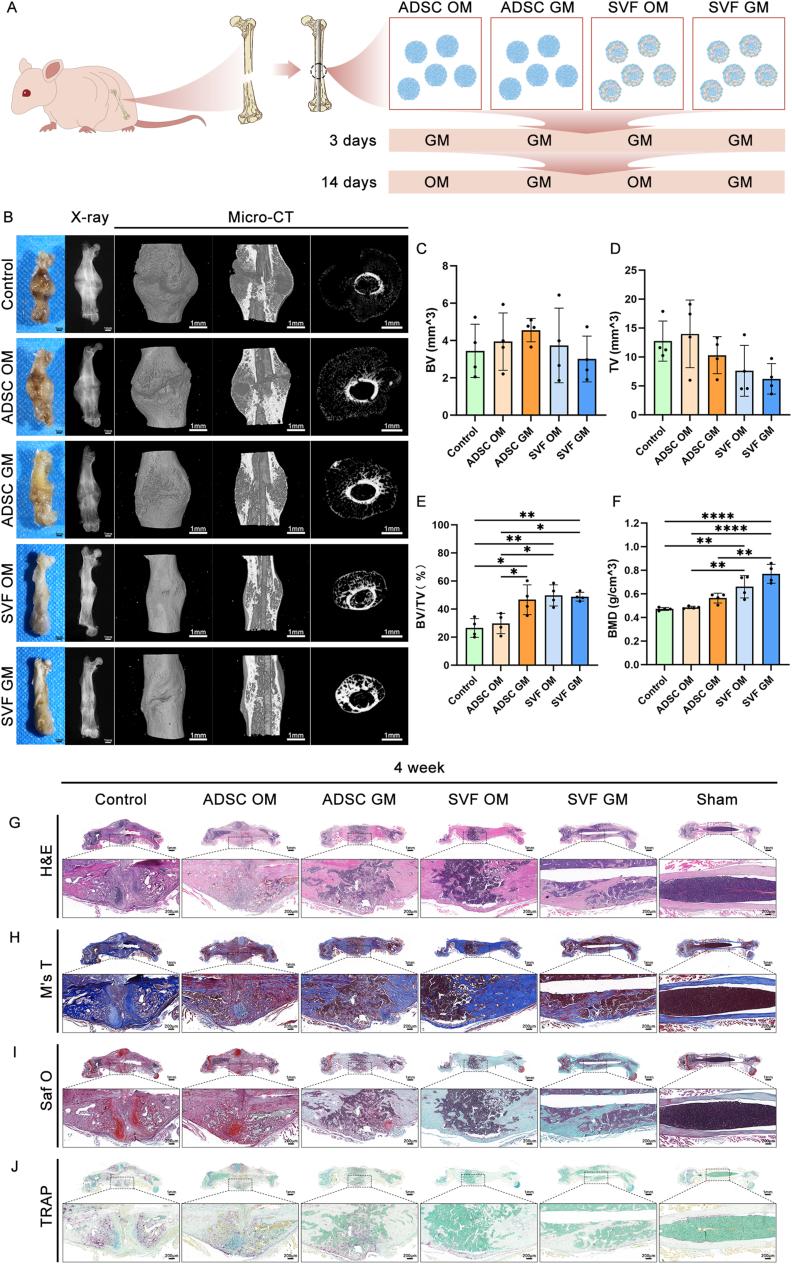
*In vivo* analysis of the SVF-GM, SVF-OM, ADSC-GM, and ADSC-OM spheroids in the treatment of femoral shaft fractures in nude mice. a) Schematic illustration of the experimental design: SVF- and ADSC-derived spheroids were cultured *in vitro* for 14 days and then transplanted into a nude mouse femoral shaft fracture model for therapeutic assessment; b) Representative macroscopic images, X-ray radiographs, and 3D reconstructed micro-CT scans (overall view, coronal plane, and transverse plane) of femora collected at week 4 post-operation. Treatment groups (from top to bottom): fracture-only control, ADSC-OM, ADSC-GM, SVF-OM, SVF-GM, and sham-operated group; c–f) Quantitative micro-CT analysis of fracture callus, including: c) Bone volume (BV); d) Total tissue volume (TV); e) Bone volume fraction (BV/TV); f) Bone mineral density (BMD); g–j) Representative histological staining images to examine bone regeneration and tissue remodeling: g) Hematoxylin and eosin (H&E) staining images; h) Masson's trichrome staining images; i) Safranin O–Fast Green staining images; j) Tartrate-resistant acid phosphatase (TRAP) staining for osteoclast activity.

Herein, we employed an immunodeficient mouse femoral fracture model to explore the therapeutic potential of SVF organoids in promoting bone fracture healing and tissue regeneration *in vivo*. Fracture sites were treated with SVF-derived organoids or ADSC spheroids cultured under either GM or OM conditions (Fig. 9A). The controls included blank (untreated) and sham-operated groups. At 4 weeks post-implantation, the mice were euthanized for macroscopic, radiographic, and histological assessment of fracture repair.

Gross morphology, X-ray imaging, and micro-CT revealed that the blank group retained a visible fracture gap, while the ADSC-GM and ADSC-OM groups exhibited partial bridging with discernible fracture lines. Conversely, the SVF-GM and SVF-OM groups showed more advanced healing, with blurred fracture lines and evident callus formation. Moreover, among all the groups, the SVF-GM group exhibited the smallest callus volume (Fig. 9B).

Quantitative micro-CT analysis of the fracture callus (excluding original cortical bone) revealed the highest BV (Fig. 9C) and smallest total TV (Fig. 9D) in the ADSC-GM and SVF-GM groups, respectively. Furthermore, the SVF-OM group exhibited the highest BV/TV, although the difference was not significant relative to the SVF-GM group (Fig. 9E). Additionally, the SVF-GM group showed the highest BMD, although the difference was also not significant compared to the SVF-OM group (Fig. 9F).

Histological analysis confirmed the above imaging findings. Specifically, according to the H&E staining results, the SVF-GM group had better callus remodeling, denser bone formation, and improved structural continuity at the fracture site (Fig. 9G), confirming the micro-CT evidence of accelerated bone regeneration. On the other hand, Masson's trichrome staining revealed more mature bone tissues in the SVF-GM group (Fig. 9H). Furthermore, Safranin O staining revealed less residual cartilage in the SVF-GM callus, suggesting a more complete bone remodeling (Fig. 9I). Moreover, according to the Tartrate-Resistant Acid Phosphatase (TRAP) staining results, the SVF-GM group exhibited the lowest number of osteoclasts at the fracture site, closely resembling normal femoral bone (Fig. 9J). In addition, hOCN staining revealed the contribution of donor cells from the ADSC-OM, ADSC-GM, SVF-OM, and SVF-GM groups to fracture healing. Notably, SVF organoids showed significantly higher hOCN expression in the fracture callus region compared to the control and sham groups. Moreover, SVF organoids outperformed ADSC spheroids in promoting hOCN expression, suggesting that SVF organoids possess better biocompatibility compared to ADSC spheroids. However, no significant difference was observed between GM and OM groups **(**Supplementary Fig. S8A–B**)**.

These findings collectively demonstrate that SVF-derived organoids could effectively promote *in vivo* fracture healing. It is also noteworthy that *in situ* implantation of SVF organoids without prior *in vitro* osteogenic induction enhanced bone maturation and accelerated fracture repair, outperforming conventional ADSC spheroid transplantation.

## 4 Discussion

Stem cell-based bone tissue engineering has recently emerged as a viable approach for treating complex orthopedic conditions such as nonunion, ON, and LBDs [11,36]. Due to ethical and safety concerns, autologous MSCs (including BMSCs and ADSCs) are increasingly gaining more attention, with ADSCs emerging as a major focus in bone tissue engineering research—particularly owing to their accessibility, robust proliferative capacity, and osteogenic potential [14,37,38].

The SVF, an adipose tissue-derived primary isolate from which ADSCs could be obtained, is a heterogeneous cell population comprising MSCs, ECs, and pericytes, among other blood-derived cells [23]. Notably, due to the compositional complexity of the SVF and phenotypic alterations that occur during adherent *in vitro* expansion [24], SVF-related research remains limited. Furthermore, although the use of the SVF in constructing vascularized adipose organoids is well-documented [7,26,27], its application in bone tissue engineering remains underexplored.

Herein, we leveraged a simple yet effective method to generate complex 3-D organoid-like structures from human adipose-derived SVF via spontaneous self-assembly. These structures met the established criteria for defining organoids, allowing us to examine how the interplay between diverse cell types could influence overall biological function. Notably, ADSC-derived spheroids served as the control, and osteoinductive and non-osteoinductive culture conditions were evaluated for their impact on biological functions *in vitro* and *in vivo*.

Compared to ADSC spheroids, SVF-derived organoids exhibited a more physiologically relevant architecture, as well as superior angiogenic, osteogenic, and immunomodulatory properties. Moreover, whether or not the SVF organoids underwent osteogenic induction *in vitro* minimally influenced their bone-healing efficacy *in vivo*, highlighting them as a readily accessible, functionally versatile cell source for bone tissue engineering that does not require extensive *in vitro* manipulation.

Organoid formation relies primarily on natural cell self-assembly processes commonly observed during embryonic development and morphogenesis, wherein cells aggregate into spheroids and interact closely with adjacent cells via intercellular junctions and adhesion molecules [39,40]. Although 3D cultures promote tight cell–cell and cell–ECM interactions that can mimic the native tissue microenvironment, diffusion could limit effective gas and nutrient exchange to a distance of ⁓200 μm. Notably, such high-density cellular structures are highly susceptible to proliferation arrest during long-term cultures and may experience central necrosis due to inadequate oxygen and nutrient supply. Consequently, vascularized organoids would be critical, especially given that vascular network integration is essential for sustaining cell viability and functional performance [[41], [42], [43]].

Since osteogenesis and angiogenesis are inherently coupled and synergistic during bone formation, the regulation of their pathways should be co-modulated in the design of optimized tissue-engineered bone constructs [44]. Nonetheless, vascularization in engineered tissues often requires the incorporation of ECs from unrelated sources. An example would be co-culturing BMSCs with Human Umbilical Vein Endothelial Cells (HUVECs) [21,22,45].

Herein, SVF organoids retained native EC components and successfully formed capillary-like networks that mimic the physiological microvasculature. Unlike conventional strategies that require exogenous Endothelial Growth Factors (EGFs) or matrix gels to promote vascularization, we primarily leveraged paracrine signaling and intercellular interactions of ADSCs to support EC survival and proliferation. Besides yielding vascular networks that more closely resemble the natural vasculature, this method also enhanced the translational potential of SVF organoids for future clinical applications.

In a previous study, ADSCs expanded through adherent culture exhibited impaired lipid metabolism and reduced capacity for small-molecule processing, which, in turn, diminished their osteogenic and chondrogenic differentiation potential relative to ADSCs within the native SVF [24]. Consistent with these findings, our induction experiments revealed that SVF possessed superior multilineage differentiation capacity. We also observed a distinct spatial organization in osteogenic, angiogenic, and chondrogenic marker expression within SVF-derived organoids. Specifically, RUNX2 was predominantly localized to the spheroid core and was closely associated with ECs, whereas ALP was primarily expressed at the periphery. Furthermore, mineralized nodules, indicative of late-stage osteogenesis, initially emerged in the spheroid core and gradually spread outward. Additionally, the vascular network was mostly concentrated in the inner regions, while SOX9, a key chondrogenic TF, was expressed in spatial proximity to RUNX2. This complex, orderly spatial arrangement aligns with Lancaster and Knoblich's definition of organoids as self-organizing, multicellular structures that mimic the physiological tissue architecture [8]. Nevertheless, additional research will be required to further elucidate the mechanisms underlying intercellular interactions and optimize culture conditions for producing more mature, vascularized osteogenic organoids.

Notably, SVF-related research has historically focused primarily on the biological functions of ADSCs and ECs, often overlooking the fact that other blood-derived cells—particularly erythrocytes and immune cells—constitute a significant portion of the SVF and may crucially modulate overall biological function [46]. Herein, we did not perform RBC lysis before culture, even though previous reports indicated that hemolysis can release toxic byproducts such as free hemoglobin and heme, which could persist in the culture environment and detrimentally affect cell health [32]. It is also noteworthy that although erythrocytes appeared not to directly participate in organoid formation, we detected various macrophage subtypes within the spheroids, primarily in the outer regions.

Due to their pivotal roles in bone repair, macrophages have recently garnered significant interest in the realm of bone tissue engineering. During the early phase of bone injury, classically activated M1 macrophages secrete pro-inflammatory cytokines, initiating an inflammatory response and thus triggering endogenous fracture healing mechanisms [28,29]. Conversely, alternatively activated M2 macrophages could release GFs that support osteoblast proliferation and differentiation, thus promoting bone regeneration during the remodeling phase [28,30]. Although the anti-inflammatory and pro-regenerative effects of M2 polarization are well-documented [47,48], it is also noteworthy that M1 macrophages may play an indispensable role during the early inflammatory phase. Specifically, M1 macrophage depletion could impair the acute inflammatory response, potentially causing delayed union or non-union bone fractures [[49], [50], [51], [52], [53]].

Herein, SVF-derived organoids exhibited significantly higher expression levels of both pro-and anti-inflammatory genes compared to ADSC spheroids. The analysis of culture supernatants and proteomic profiling further revealed that SVF organoids were enriched in both immune regulatory pathways and cytokine signaling functions. Compared to ADSC spheroids, SVF organoids demonstrated more diverse and dynamic secretory profiles, which substantially enhanced their functional potential in immunomodulation, tissue repair, and inflammatory response. In subsequent inflammatory stimulation experiments, SVF organoids displayed a time-dependent immunoregulatory pattern: early upregulation of pro-inflammatory markers was followed by a gradual decline over time, whereas anti-inflammatory markers progressively increased. Additionally, SVF organoids exhibited a unique spatially dependent bidirectional immunomodulatory response. Specifically, anti-inflammatory gene expression was markedly upregulated in the spheroid core, while pro-inflammatory markers were predominantly expressed at the periphery. This distribution pattern is highly reminiscent of the immune microenvironment in native tissues, where pro- and anti-inflammatory responses complement each other to maintain immune homeostasis. These findings suggest that SVF organoids may possess tissue-level immunoregulatory capacity to maintain homeostasis within complex tissue microenvironments. This highlights their strong translational potential in inflammation-related disease models, personalized therapies, and regenerative medicine. Nonetheless, additional research will be required to further elucidate the precise mechanisms through which this spatial immunomodulation contributes to tissue repair *in vivo*.

Despite its valuable insights, this study has several notable limitations. First, both SVF and adherent-expanded ADSCs exhibited a degree of donor-dependent heterogeneity, leading to variabilities in the size of the resulting organoids and in gene expression profiles despite the initial seeding cell number being standardized. Therefore, larger-sample studies will be required to further validate the findings’ statistical power and scientific reliability. Nevertheless, this heterogeneity highlights the potential of SVF-derived organoids as patient-specific *in vitro* disease models for drug screening and tailored therapeutic applications.

Second, we did not explore the presence or functional roles of other immune cell types within SVF organoids, such as T cells, B cells, and neutrophils. Therefore, in addition to comprehensively characterizing the immune cell composition of SVF organoids, future studies should also elucidate their immunoregulatory mechanisms both *in vitro* and *in vivo.*

Third, cytokine profiling of culture supernatants and proteomic analysis of spheroids were performed only as a cross-sectional snapshot at day 14 post-formation, highlighting the need for longitudinal analyses across multiple time points to better understand dynamic tissue development and maturation.

Fourth, non-specific signals in necrotic regions—particularly in the central zones of ADSC spheroids—could have affected the IF staining results. Although we confirmed nuclear co-localization of fluorescent signals to mitigate this phenomenon, residual non-specific expression could still have introduced some bias. Therefore, for more comprehensive and reliable results, an integrated approach involving multi-layered analyses—such as transcriptomics, proteomics, and histological assessments—will be required.

Fifth, the engineered SVF-derived organoids still diverged considerably from native bone tissue; hence, future studies should modify both the molecular and biomechanical microenvironment, potentially optimizing culture conditions and yielding organoids with improved vascularization and enhanced osteogenic performance.

In recent years, bone organoids have demonstrated increasing potential for applications in regenerative medicine and clinical translation. Studies have shown that bone organoids not only recapitulate the structural and functional features of native bone tissue but can also serve as platforms for disease modeling and personalized therapeutic interventions [54,55]. For example, Su et al. reviewed recent breakthroughs that integrate large-scale self-mineralizing bone organoids with 3D bioprinting technologies. These constructs exhibit multi-lineage differentiation, self-organization, and mechanical integrity, and have shown promising outcomes in in vivo models of bone defect repair [56]. These advances indicate that bone organoid technologies are steadily transitioning from basic research to clinical application, offering new avenues for treating complex bone disorders.

## 5 Conclusion

Herein, we developed a structurally complex organoid using adipose tissue-derived SVF and conducted a comprehensive comparative analysis with adherent-expanded ADSCs. Additionally, we systematically evaluated the *in vitro* and *in vivo* biological effects of both the GM and OM conditions.

According to the results, GM-cultured SVF spheroids exhibited the most physiologically relevant architecture and sustained proliferative capacity. Furthermore, compared to ADSCs, SVF organoids displayed superior osteogenic, chondrogenic, and adipogenic differentiation potential. Additionally, secretome profiling via protein microarray and proteomic analysis of the spheroids revealed enhanced inflammatory regulation and cytokine signaling capacities in SVF spheroids. Moreover, compared to those cultured in OM, GM-cultured spheroids exhibited greater ECM synthesis, as well as enhanced cell proliferation and migration.

*In vivo* femoral fracture repair experiments further revealed that SVF spheroids significantly accelerated bone healing compared to ADSC spheroids. Moreover, the spheroids pre-cultured in growth and osteogenic media showed no significant differences, suggesting that osteogenic pre-induction may not be required for effective bone regeneration *in vivo*.

Overall, SVF-derived organoids represent a promising strategy for developing advanced cell-based therapeutics that could be used to treat complex orthopedic conditions such as nonunion, ON, and LBDs.

## CRediT authorship contribution statement

**Jiazhou Wu:** Writing – original draft, Visualization, Investigation, Formal analysis, Data curation, Conceptualization. **Ying He:** Methodology, Formal analysis, Data curation, Conceptualization. **Tao Qian:** Software, Methodology, Formal analysis, Data curation, Conceptualization. **Zexian Liu:** Validation, Methodology, Formal analysis, Data curation. **Yanbin Wu:** Resources, Conceptualization. **Yazhou Li:** Visualization, Validation, Supervision. **Hongyu Jiang:** Supervision, Resources, Investigation. **Jianting Ye:** Software, Resources, Funding acquisition. **Jia Lv:** Supervision, Software, Resources. **Biao Ma:** Visualization, Validation, Supervision. **Endong Luo:** Visualization, Software, Methodology. **Jialiang You:** Resources, Methodology, Investigation. **Dingkai Wang:** Resources, Methodology, Conceptualization. **Yun Bai:** Software, Data curation, Conceptualization. **Junming Zhang:** Resources, Methodology. **Liang Zuo:** Methodology, Conceptualization. **Jiang Peng:** Writing – review & editing, Software, Resources, Project administration, Funding acquisition, Conceptualization.

## Ethics approval and consent to participate

This study involving human participants was approved by the Ethics Committee of the Chinese PLA General Hospital (Approval No. 2023KY088-KS001). Adipose tissue samples were collected from 50 male patients (aged 20–50 years; mean age: 34.96 years; BMI: 26.06 ± 3.84 kg/m^2^) who underwent liposuction at the Fourth Medical Center of the Chinese PLA General Hospital. All participants provided written informed consent prior to inclusion in the study. None of the participants had a history of metabolic or endocrine disorders, alcohol abuse, smoking, or use of medications affecting glucose or lipid metabolism. The study was conducted in accordance with the Declaration of Helsinki and relevant institutional and national guidelines.

All animal experimental procedures were approved by the Animal Welfare and Ethics Committee of Zhongyan Zichuang (Beijing) Biotechnology Co., Ltd. (Approval No. ZYZC202404019S), and conducted in accordance with institutional and national guidelines for the care and use of laboratory animals.

## Declaration of competing interest

The authors declare no competing financial interests.
