# Supplementary material for: Stromal vascular fraction self-assembles vascularized osteogenic organoids with immunomodulatory functions
Source: Bioact Mater. 2025 Nov 14;57:323–43. doi: 10.1016/j.bioactmat.2025.10.030 (PMC12662011; doi:10.1016/j.bioactmat.2025.10.030)
Supplement: Multimedia component 1 [file mmc1.docx]

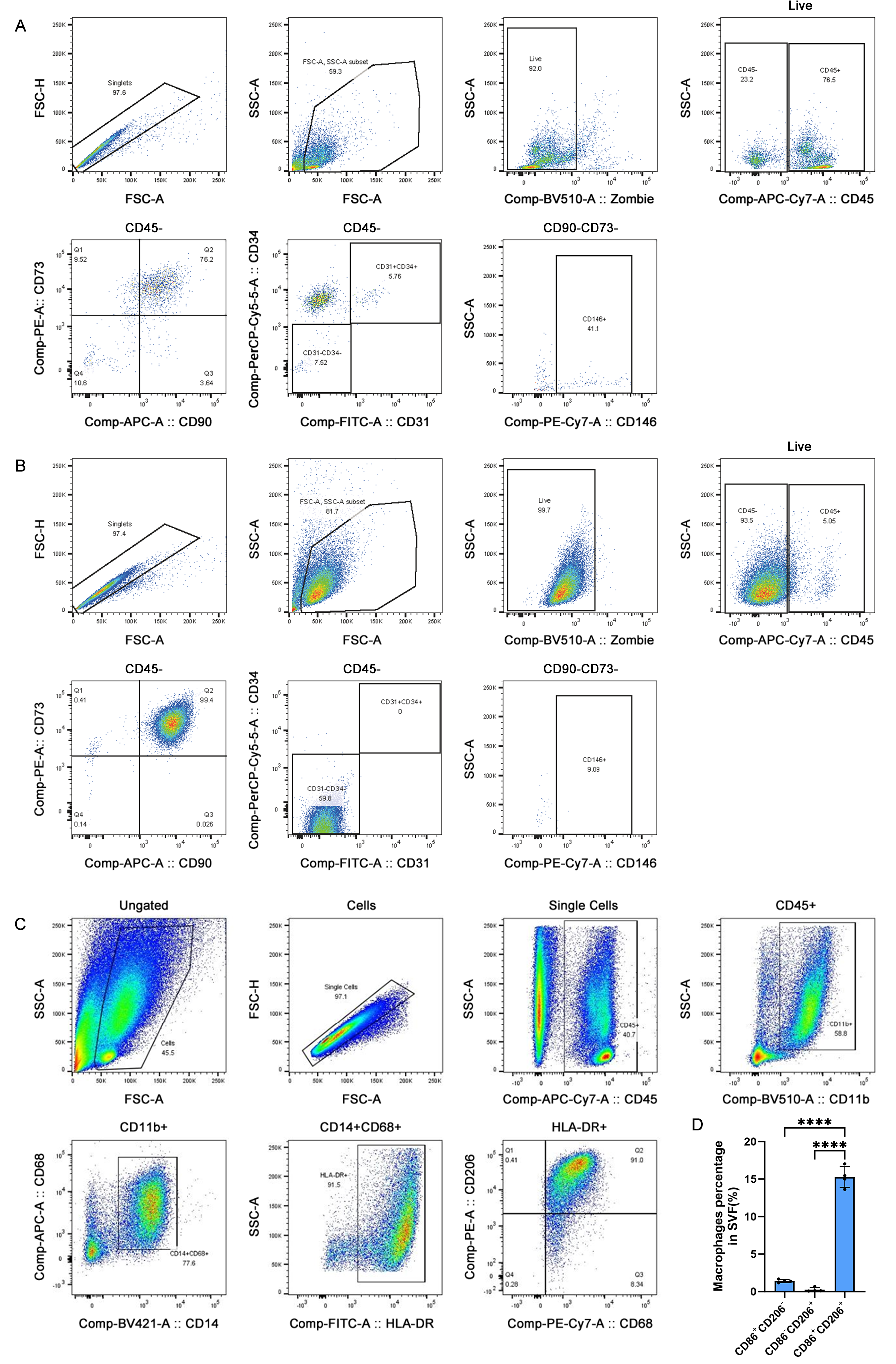


**Supplementary Figure. S1.** Phenotypic analysis of SVF and P0 adipose-derived stem cells (ADSCs) by flow cytometry. a) Representative flow cytometry plots of primary adipose-derived mesenchymal stem cell (P0 ADSC) subpopulations; b) Representative flow cytometry plots of freshly isolated stromal vascular fraction (SVF) subpopulations; c–d) Representative flow cytometry plots and quantitative analysis of macrophage subtypes within freshly isolated SVF.


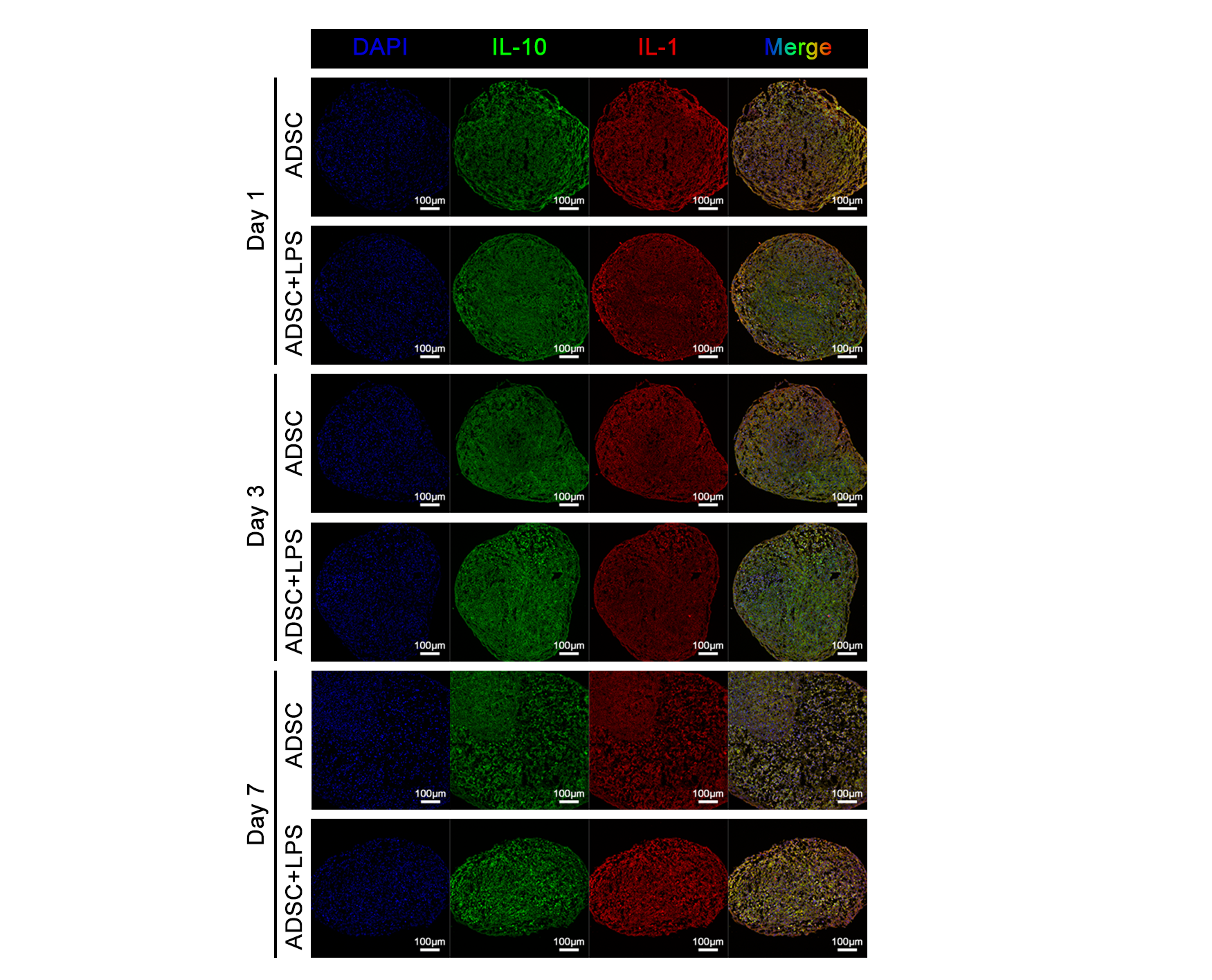


**Supplementary Figure. S2.** Inflammatory response analysis of ADSC spheroids. Representative multiplex immunofluorescence images of ADSC spheroids at days 1, 3, and 7 after LPS stimulation, showing co-localization of key inflammatory markers: nuclei (DAPI), IL-10 (anti-inflammatory cytokine), IL-1 (pro-inflammatory cytokine), and merged channels.


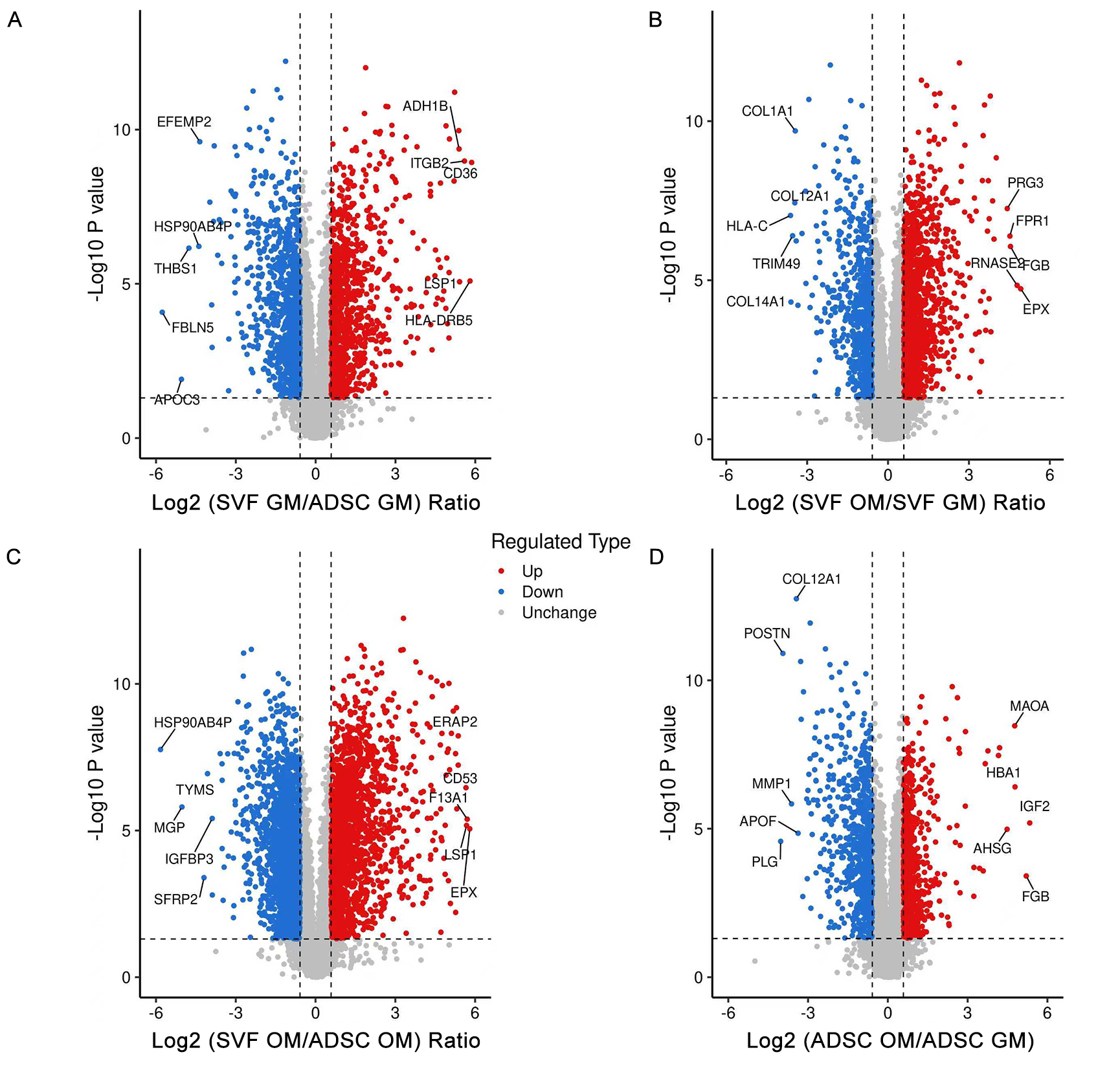


**Supplementary Figure. S3.** Differential protein expression analysis among SVF-GM, SVF-OM, ADSC-GM, and ADSC-OM groups using volcano plots. a) Volcano plot of SVF-GM vs. ADSC-GM; b) Volcano plot of SVF-OM vs. SVF-GM; c) Volcano plot of SVF-OM vs. ADSC-OM; d) Volcano plot of ADSC-OM vs. ADSC-GM. Each plot displays the statistical significance (P value) versus fold change (log₂ scale) for all detected proteins.


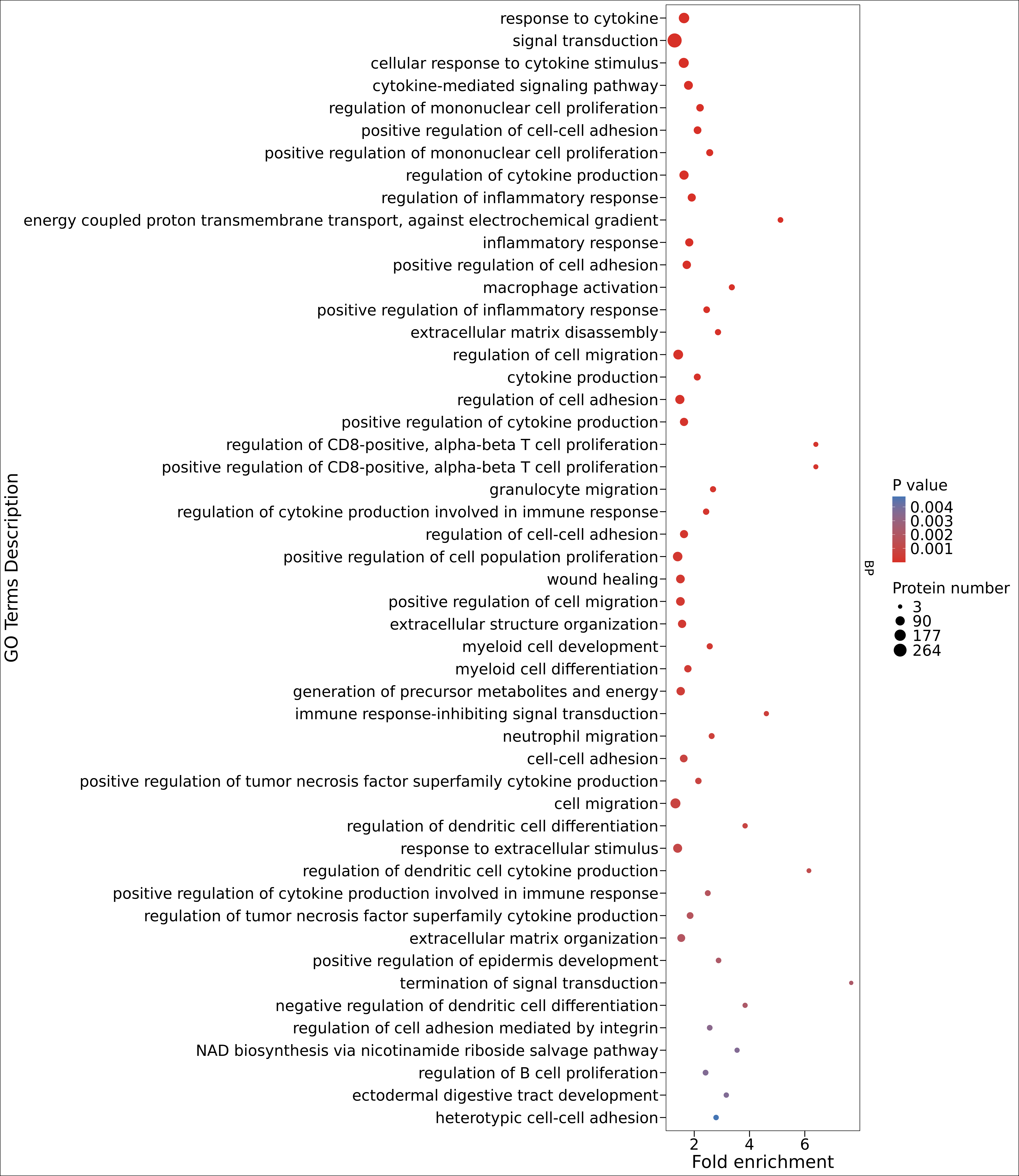


**Supplementary Figure. S4.** The top 50 significantly enriched Gene Ontology Biological Process (GO-BP) terms between SVF-GM and ADSC-GM.
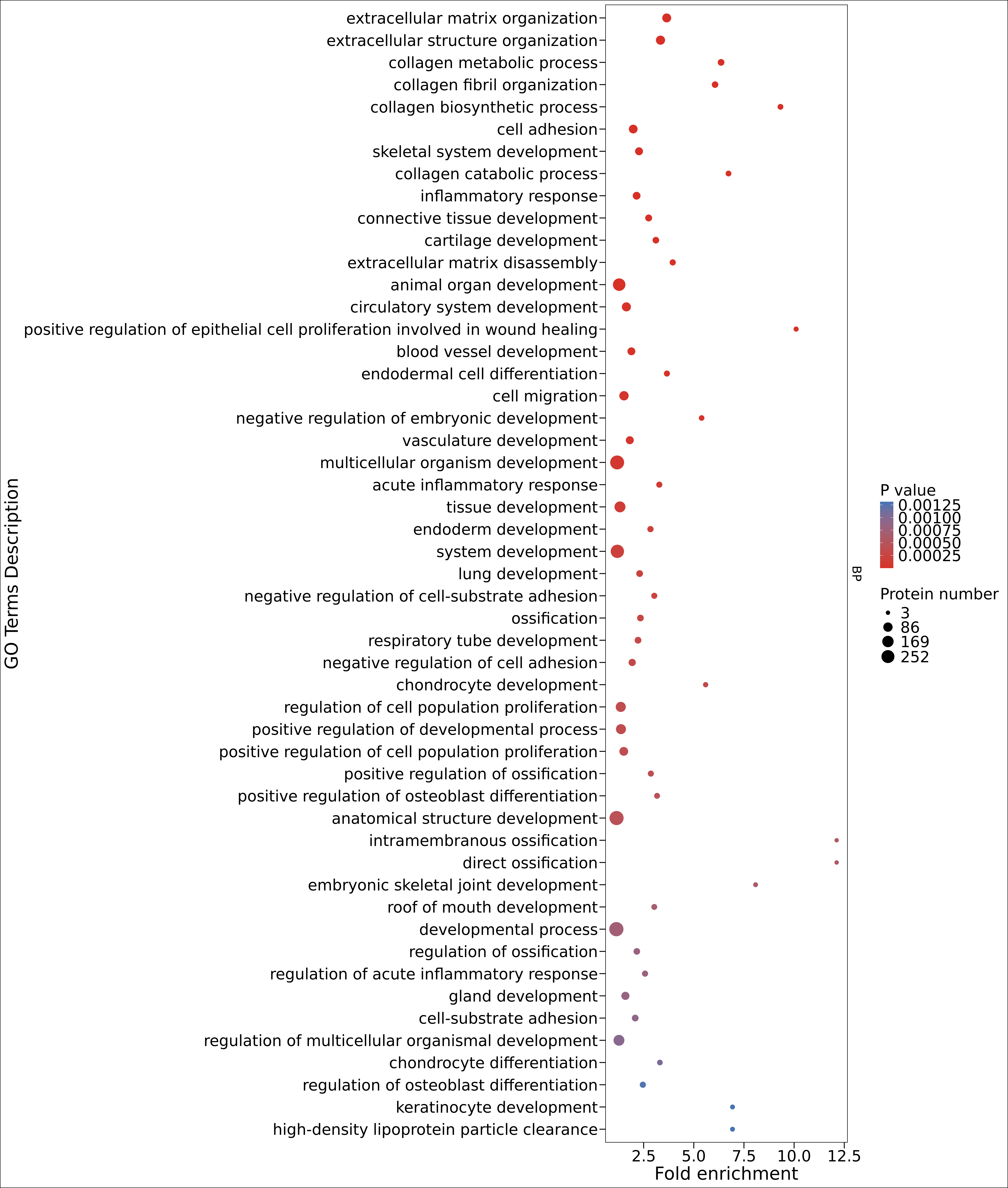


**Supplementary Figure. S5.** The top 50 significantly enriched GO-BP terms between SVF-GM and SVF-OM.
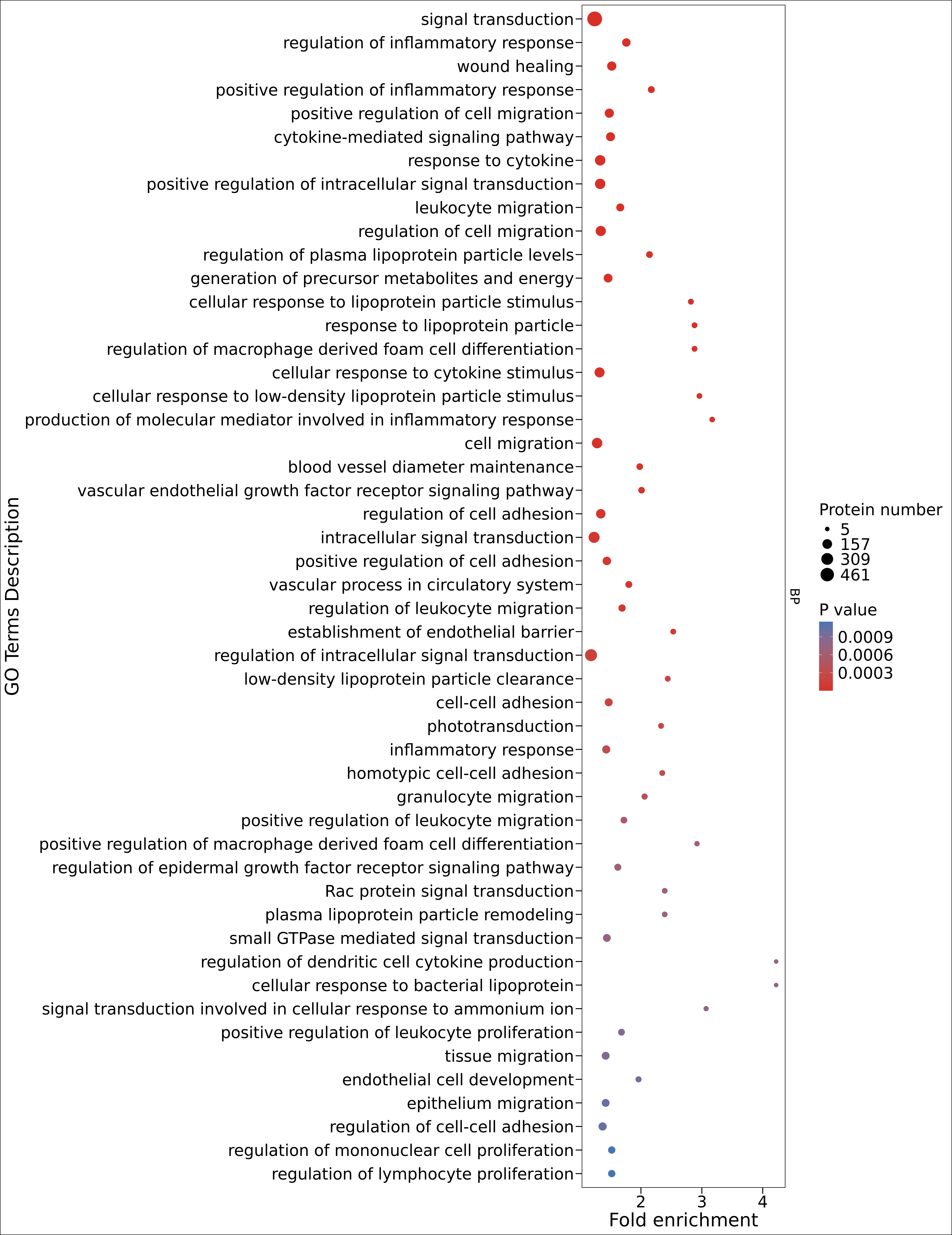


**Supplementary Figure. S6.** The top 50 significantly enriched GO-BP terms between SVF-OM and ADSC-OM.
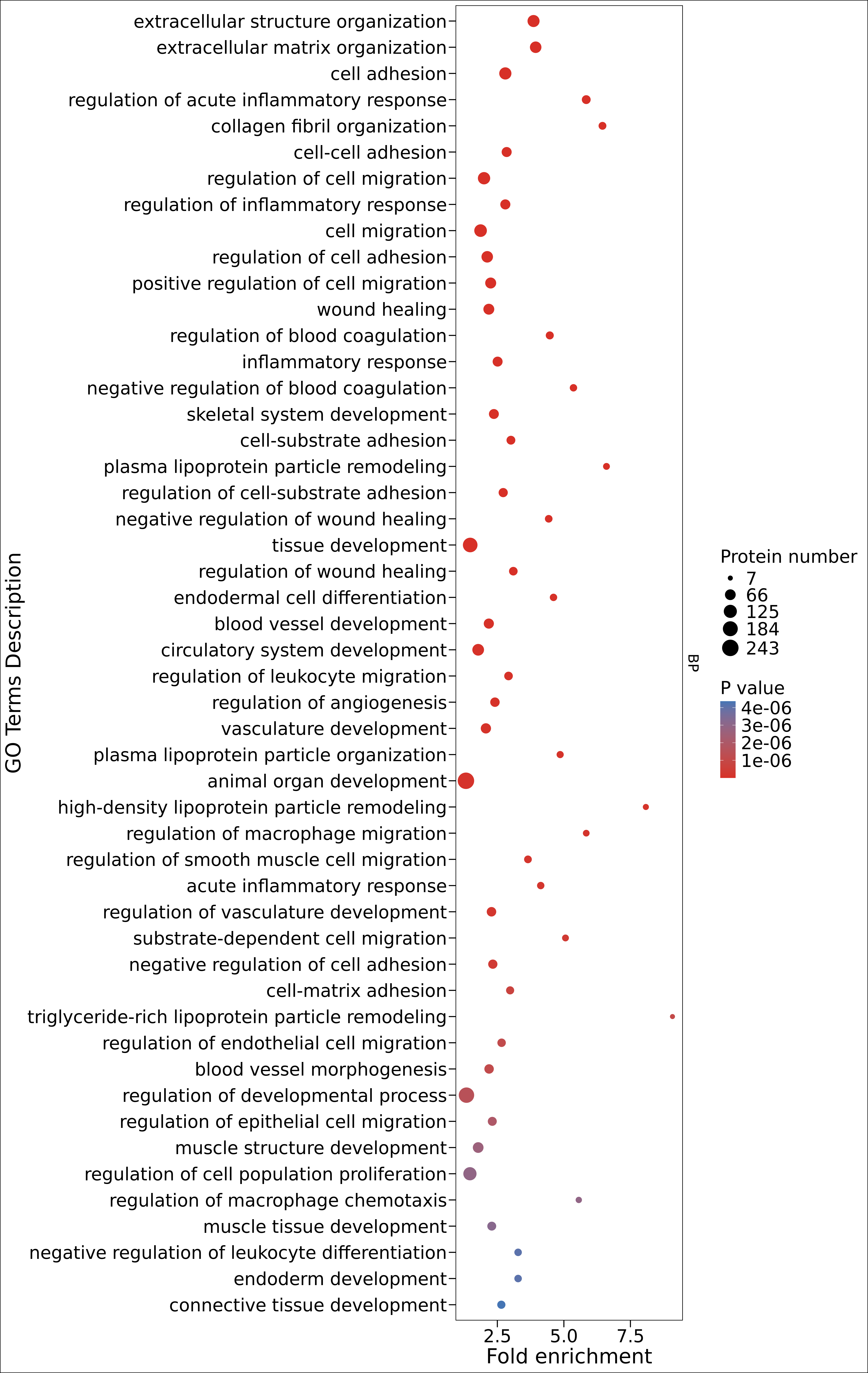


**Supplementary Figure. S7.** The top 50 significantly enriched GO-BP terms between ADSC-GM and ADSC-OM.


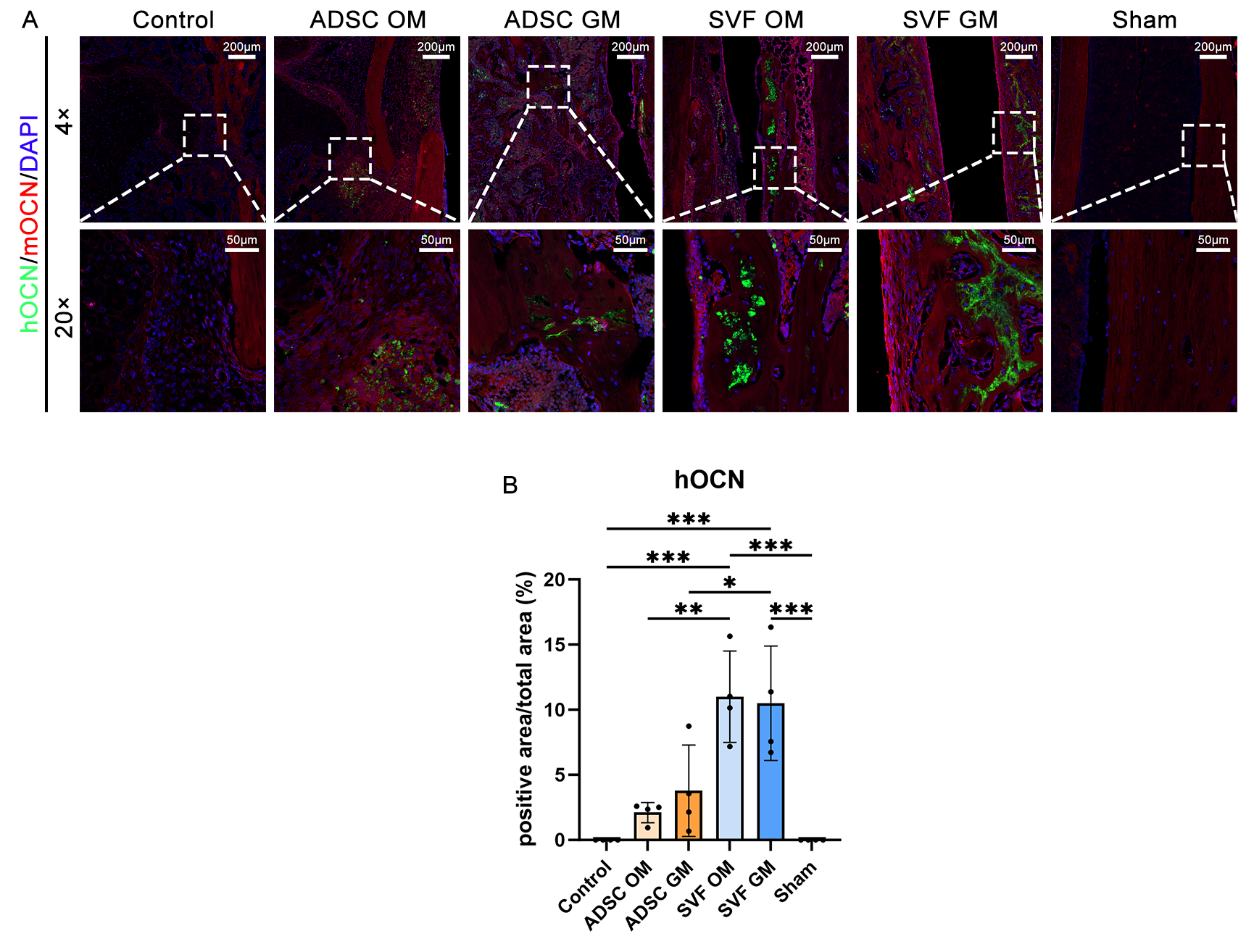


**Supplementary Figure. S8.** In vivo biocompatibility analysis of ADSC-OM, ADSC-GM, SVF-OM, and SVF-GM spheroids in a nude mouse femoral fracture model. a) Representative merged immunofluorescence images at week 4 post-operation, showing human osteocalcin (hOCN, green), mouse osteocalcin (mOCN, red), and nuclei (DAPI, blue). Images are shown from left to right for the following groups: fracture-only control, ADSC-OM, ADSC-GM, SVF-OM, SVF-GM, and sham-operated group; b) Quantitative analysis of hOCN-positive area proportions to compare expression levels among different groups.
